# An application of Bayesian Belief Networks to assess management scenarios for aquaculture in a complex tropical lake system in Indonesia

**DOI:** 10.1371/journal.pone.0250365

**Published:** 2021-04-16

**Authors:** Ivana Yuniarti, Klaus Glenk, Alistair McVittie, Sulung Nomosatryo, Endra Triwisesa, Tri Suryono, Arianto Budi Santoso, Iwan Ridwansyah

**Affiliations:** 1 School of Geosciences, University of Edinburgh, Edinburgh, United Kingdom; 2 Department of Rural Economy, Environment and Society, Scotland’s Rural College, Edinburgh, United Kingdom; 3 Research Centre for Limnology, Indonesian Institute of Sciences, Cibinong, Indonesia; 4 GFZ German Research Centre for Geosciences, Section Geomicrobiology, Potsdam, Germany; Swedish University of Agricultural Science, SWEDEN

## Abstract

A Bayesian Belief Network, validated using past observational data, is applied to conceptualize the ecological response of Lake Maninjau, a tropical lake ecosystem in Indonesia, to tilapia cage farms operating on the lake and to quantify its impacts to assist decision making. The model captures ecosystem services trade-offs between cage farming and native fish loss. It is used to appraise options for lake management related to the minimization of the impacts of the cage farms. The constructed model overcomes difficulties with limited data availability to illustrate the complex physical and biogeochemical interactions contributing to triggering mass fish kills due to upwelling and the loss in the production of native fish related to the operation of cage farming. The model highlights existing information gaps in the research related to the management of the farms in the study area, which is applicable to other tropical lakes in general. Model results suggest that internal phosphorous loading (IPL) should be recognized as one of the primary targets of the deep eutrophic tropical lake restoration efforts. Theoretical and practical contributions of the model and model expansions are discussed. Short- and longer-term actions to contribute to a more sustainable management are recommended and include epilimnion aeration and sediment capping.

## Introduction

Inland water cage culture farms (IWCCF) operate at a range of spatial scales, from traditional households to corporate modern farms. IWCCF is rapidly growing around the globe as an answer to the increasing demand for aquatic products [1]. In Asia, cage culture is one of the most widely practised aquaculture systems in terms of both production and value [2, 3]. The rapid development of cage culture in the region is often attributed to the necessity to provide fish as an affordable nutritional source and to generate employment opportunity, both in support of poverty alleviation efforts [4–6].

Despite their benefits, there are increasing concerns about the negative impacts of IWCCF on the aquatic environment. One of the widely identified effects is eutrophication [2, 7, 8]. Eutrophication leads to blooming algae and the degradation process of the algae consumes dissolved oxygen (DO). This process then leads to DO depletion causing mass fish kills (MFK) [9, 10]. Other impacts are changes in macro and microbenthic population, and effects on fish biodiversity [11, 12].

MFK events have been long reported from various lakes, rivers, and reservoirs in Indonesia and many other countries (see [13–17]) and they have caused huge financial loss [18]. This raises the need to provide a model that can predict MFK to mitigate the loss. However, data limitations can sometimes hinder the development of mechanistic models. Thus, engaging with approaches such as Bayesian Belief Networks (BBN) becomes an interesting alternative. Two papers have used BBN to predict algal blooms related to several nutrient input scenarios (see [19, 20]). Both studies were conducted in temperate lakes in high income countries where monitoring data of nutrient inputs are reasonably adequate. They use a relatively low number of nodes (maximum of nine) to account for the principal drivers of algal blooms. However, we are not aware of any studies that use BBN to predict the MFK in deep tropical lakes with complex and specific ecological characteristics [21, 22].

In this study, we apply BBN to generate information on the ecological response to IWCCF that can underpin appraisals of IWCCF management scenarios of Lake Maninjau, Indonesia. We specifically aim to answer the research question: to what extent can BBN be used to conceptualize the lake’s complex ecological system related to IWCCF management and quantify its impacts to assist decision making? We surmise that BBN is particularly suited to investigate the impacts of IWCCF on the occurrence of MFK incidents due to upwelling processes in locations where data is scarce. The developed model can then help improve our understanding of both the economy-environment trade-offs of IWCCF expansion and the effects of IWCCF and upwelling processes on a native fish species, *Gobiopterus* sp. This species is the only finfish species reported by the fishers to diminish during and after major upwelling events; thus, it is chosen as the indicator of IWCCF impacts on the native biota.

This study differs from previous BBN applications [19, 20] because it includes more complex relationships between physical, biological, and chemical components of the lake ecology, in addition to human activities–IWCCF management. Thus, it better illustrates the complexity of the ecosystem. Moreover, this study emphasizes the advantage of BBN in overcoming limited data, which is a common problem in the Global South. Through the development and testing of the BBN in our case study, we are able to identify important gaps in data in the studied lake and beyond that should be addressed to improve model accuracy and facilitate development of mechanistic models. We apply the BBN to investigate a range of management intervention scenarios. This provides important information to inform sustainable lake management.

Beyond the applied perspective, the paper makes several contributions to the academic literature. First, we develop an integrated model to predict the probability of MFK incidents in a complex tropical lake system. To the best of our knowledge, this is the first such modelling effort. In line with this, [23] identified that BBN application in tropical region is limited. Second, this paper contributes to the growing literature on the use of BBN to assess ecosystem service (ES) trade-offs; this paper specifically investigates trade-offs between two provisioning services: fish provided by aquaculture and a native species capture fishery. There are few BBN case studies that aim to simulate ES trade-offs especially in complex-spatial systems [23–25]. More importantly, this study rigorously applies a variable correlation table to build the direct acyclic diagram (DAG), which is rarely conducted by previous BBN studies [26]. The applied and methodological contributions highlighted above carry insights that may be used in different lakes across the tropics that are subject to planned or ongoing IWCCF.

The paper is organised as follows: first, a brief overview of the history of IWCCF in Lake Maninjau and of the lake’s current ecological condition. An introduction of the BBN approach follows this. The third section will present the methodology applied to build and validate the model. In the fourth section, the developed model is shown and the results of several IWCCF management scenarios are presented. A concluding section then follows a discussion of model performance and scenario results.

### Site description

**Fig 1:**
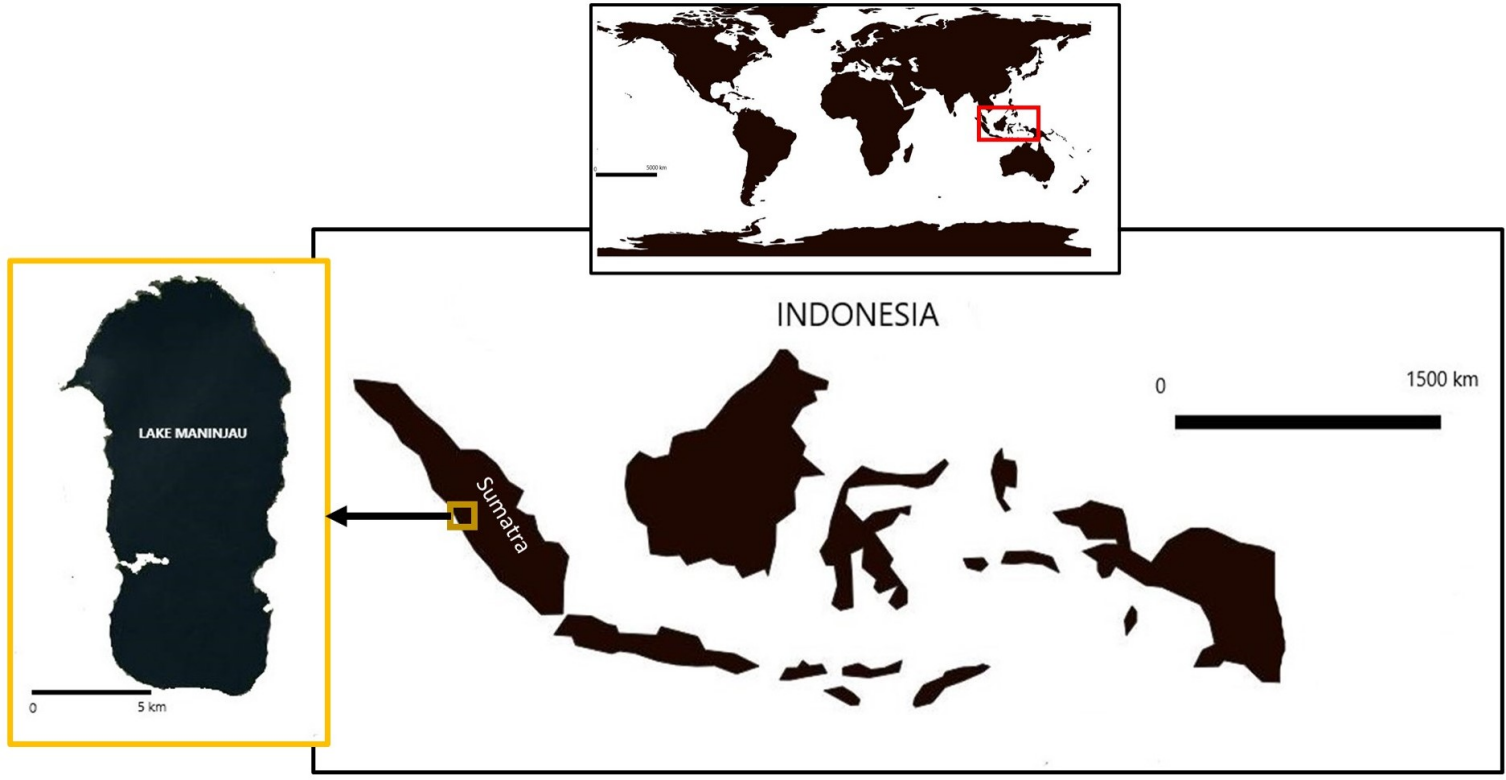
Study site. (Source: Authors’ creation based on maps provided by http://www.naturalearthdata.com/, retrieved on 2 March 2021.) The world map is similar to but not identical to the original image and is therefore for illustrative purposes only.

Lake Maninjau (Fig 1) is one of the big volcanic lakes in Sumatra Island, Indonesia. Its total surface area is 97.37 km^2^, and its catchment area is approximately 132.6 km^2^ with a full water volume of 10 million m^3^ [27]. Its geographical positions are 100^o^ 08’5384” E to 100^o^ 14’02.39” E and 0^o^ 14’ 52.50” S to 0^o^ 24’ 12.17” S.

It is selected as the case study as it represents many Indonesian lakes formed by a volcanic and or tectonic processes in complex tropical systems [28]. Because of its geological formation, the lake has a specific character. It is naturally rich in sulphur, which may be deposited in sediment [29] and drives its ecological process [10, 29]. Therefore, it has a natural sulphur purification activity locally named as *tubo belerang* [30]. Thus, these type of lakes are generally considered to be inappropriate for IWCCF practices from an ecological perspective [31].

The sulphur cycle interrelated with organic material is the primary key to balance this lake’s ecosystem and should be preserved to prevent severe consequences such as MFK. The balance is produced by an oxidation process depending on oxygen level and phototropic sulphur bacteria activities. Sulphide, which is a reductive form of useful sulphate or elemental sulphur, actually can be easily oxidized to elemental sulphur if there is oxygen or the mentioned bacterial activity [29]. In sufficient DO condition, the sulphide will react with reactive iron ions (Fe^2+^) creating FeS compounds. FeS will the react to form pyrite sulphide (FeS_2_), a stable structure which can sink to the sediment. However, in the condition when Fe^2+^ is limited, more hydrogen sulphide (H_2_S) is unbounded and accumulated in pore waters [32]. Further, reactive Fe (Fe^2+^) is oxidised into Fe^3+^, which will bind phosphate ions (PO_4_^3-^) to form FePO_4_ compounds. However, [10] and [29] elucidated that high H_2_S can be produced in the hypolimnion if there is a high rate of organic material degradation. In this circumstance, limitation of free reactive Fe will boost reduction of Fe^3+^ to Fe^2+^ to form FeS, but it will unbind the phosphate ions with the help of sulphate reduction microbes. As a result, the phosphate ions are released back into the hypolimnion, which is then diffused to the metalimnion and epilimnion, where the phosphate ions contribute to eutrophication.

As for many lakes in Indonesia (and in the Global South in general), there is limited (time series) data available for Lake Maninjau concerning important biogeochemical indicators and general lake condition. The Research Centre for Limnology, Indonesian Institute of Sciences (RCL-IIOS), has collected data on selected limnological aspects over the years. However, the data was sporadically collected due to financial constraints and is therefore patchy [33]. Because these data gaps exist, a mechanistic model illustrating the connection between IWCCF management and its results on the water quality improvement has not yet been developed. We consider a BBN to be a suitable alternative to fill this gap.

Biologically, the lake is the habitat for 18 types of finfish, which become the target of capture fishery [34]. Among the identified species, *Rasbora maninjau* and *Gobiopterus* sp. are the most economically important. Importantly, [35] described *Rasbora maninjau* as an endemic species living only in this lake.

IWCCF in the lake, introduced in 1992, has become the main source of livelihood for the locals. By 2015, reportedly 23,566 cages were operated in the lake [36]. In 2016 there were 1,636 registered IWCCF owners, 146 cage operators, 1,383 feed producers, and 26 feed distributors [37]. IWCCF has been estimated to generate a financial value of 12 million USD per year [38].

However, IWCCF operation in the lake has also been reported to be the cause of a severe decrease in water quality, which is indicated by eutrophication and more frequent MFK incidents [15, 39–41]. An MFK taking place in 2018 was estimated to result in a financial loss in excess of 8.7 million USD [42].

Excessive nutrient input from accumulated feed waste correlated with the use of DO for decomposition is further related to the DO depletion. This process is associated with widening anoxic layer which becomes the catalyst for more frequent MFK events. The thermocline was observed in the depth of 4–20 m [43]. They recognized that the anoxic layer was found at this depth. As a consequence, a strong wind can trigger the mixing and bring the chemical substances in the anoxic layer to surface layer. The combination of DO deprivation and H_2_S released during the upwelling process are lethal to the fish [10]. Furthermore, data collected by the authors in 2019 also shows that *Gobiopterus* sp. could not be found for months after the MFK incidents [44]. The implication of this situation is income loss for local fishers and forgoing one of the affordable protein sources for the locals.

### Bayesian Belief Networks (BBN)

A BBN is a graphical interface presenting a joint probability of various variables which can link actions to outcomes [45–48]. Mostly, it is used in Decision Support System (DSS) implementing Bayes’ rule on probability (Eq 1) [49]. Increasing applications in environmental risk assessment is also documented [50–52]. Other applications as a tool for understanding complex (socio-ecological) systems, habitat suitability, management evaluation, and modelling ES are well reported [23, 25, 53–57]. Further, BBN are also increasingly applied in environmental studies to connect management actions and environmental impacts in complex environments using interdisciplinary knowledge (i.e., [57–59]).

$$P(H|E)=\frac{P(H)\times P(E|H)}{\int P(H)\times P(E|H)dE}$$ (1)

Note: P(H) = the probability of event H will be in a particular state

P(E│H) = the conditional probability of E given H

P(H│E) = the updated probability of event H will be in a particular state given that evidence E is happening

The use of BBN as a modelling tool in interdisciplinary research is related to its flexibility to effectively combine various multidisciplinary perspectives [48]. Further, BBN as a modelling tool allows connecting a data learning process and expert knowledge. This flexibility makes it very useful to address research questions considering existing gaps in empirical data [46, 60].

BBN may encourage communication between scientists and decision-makers, who may have a limited background in the specific science knowledge [61]. Another merit is the possibility to relate BBNs to a participatory decision-making process [62, 63] to combine both empirical data and expert knowledge to address challenges associated with data and evidence gaps [45, 64]. However, [65] advised to carefully validate the constructed model if it is built based on expert domains solely.

Despite the advantages of BBN, one of the limitations is that they cannot incorporate and model feedback loops [66]. Another limitation is the difficulty to capture dynamic processes [23, 60]. Further details about the advantages and limitations of the model are discussed by [24].

## Materials and methods

### Ethical clearance

The research has been granted an ethical clearance from the ethic committee of the School of Geosciences, University of Edinburgh.

### Conceptual model development

The key variables influencing upwelling events and MFK were determined by referring to peer-reviewed literature (S1 Table in S1 File). This approach was also used by [67]. Thirty-five selected variables comprising physical, chemical, biological, and anthropogenic aspects were selected. An initial version of a variable correlation table was created to avoid over-complexity. The collaborators from RCL-IIOS then reviewed the table. The use of the variable correlation table made the process of incorporating the contributors’ advice become much easier. The final influence table is shown in S2 Table in S1 File. An acyclic graph was built referring to this final correlation table. Prior corresponding conditions were used as the principal design, referring to expert knowledge and literature as presented in the table.

### Software and data availability

- Netica software 6.05 (Norsys Software Corp, 2019). First available: 1995. Cost: Educational/personal: 285 USD. Contact: info@norsys.com
- R-lake analyser 1.11.4.1 (GLEON.org, 2019). First available: 2011. Cost: free
- Stata 15 (Timberlake Consultant Limited, 2018). First available. Cost: 355 USD
- Data availability: Data used in this paper is available in the S2 and S4 Tables in S1 File. Other data are available online at https://github.com/ivanayuniarti/BBN.

### BBN construction and model parameterisation

The directed acyclic graph (DAG) was used to construct the model in Netica software (Norsys Software Corp, 2019). The states were set based on literature described in Table 1 and limited to not more than five states to keep the model simple. In cases where there were more than five states, such as for the wind speed node and for wind direction, these were reduced to a maximum of five states (see [65]). A loop problem occurred when defining the network. The loop comprised light intensity, water transparency, DO epilimnion, and Chlo-a epilimnion. Actually, light intensity is directly correlated to photosynthesis capacity and reproductive ability of the algae before it is further related to the production of oxygen. As a solution to the problem, light intensity was connected directly to DO epilimnion instead of Chlo-a epilimnion to represent photosynthesis process.

**Table 1:** Engaged methods in BBN construction and parameterisation.

Parameters	Node	Type of Node	Method to fill CPT Tables	Data Source (s)	Discretization methodology	States
Physic nodes	Season	Parent	Data	BMKG data (2000–2010)	Classification in BMKG	Dry; Wet
	Rainfall intensity	Child	Data	BMKG data (2000–2010) (n = 3,650)	Classification in BMKG	No rain:0–1mm; Light rain:1–1mm; Moderate rain:5–10mm; Heavy rain:>10mm
	Cloudy	Child	Data	BMKG data (2000–2010)	Classification in BMKG	Yes, No
	Light intensity	Child	Data	Based on solar radiation data from BMKG (2000–2010)	Expert knowledge	Low; medium; high
	Windspeed	Child	Data	BMKG data (2000–2010) (n = 3,650)	Simplified Beaufort wind speed scale	Calm:0–19 km/hour; Moderate:20–29 km/hour; Strong: 30–50 km/hour; Gale–Storm:>50 km/hour
	Mixing	Child	Expert knowledge	Based on criteria of environmental condition to trigger upwelling from Ministry of Marine and Fisheries verified with data from Fisheries Agency and RCL-IIOS	Expert knowledge recalibrated with sensitivity to parameter (to MFK)	Yes, No
	Wind Direction	Parent	Data	RCL-IIOS data (2018) (n = 10,926)	Beaufort scale	North; North East; East; South East; Other directions
	Water transparency	Child	Data	RCL-IIOS and [33]	[69]	Low: ≤ 2 m; High:> 2 m
	Anoxic layer	Child	Expert knowledge	Based on data RCL-IIOS’s dissolved oxygen data (2001–2014)	Expert knowledge	Wide; Narrow
	Epilimnion zone after mixing	Child	Expert knowledge		[70]	Not sufficient DO:0–1.5 mg/l; Sufficient DO:>1.5 mg/l
	SSI	Child	Expert knowledge	Based on trend of calculated SSI from high frequency measurement data by RCL-IIOS (2014–2017) (n = 540)	[68]	High; Medium; Low
	Water current velocity	Child	Data	RCL-IIOS combined with data from BMKG		Fast; medium; Slow
	Anoxic hypolimnion	Child	Expert knowledge	Based on [10, 71]	[10]	Yes, No
Chemical and biological nodes	BOD epilimnion	Child	Expert knowledge	Based on patchy data from [72, 73]]	[69]	High: ≥3 mg/l; Low: <3 mg/l
	PO4 Concentration Epilimnion	Child	Expert knowledge	Based on patchy data of Total Phosphate data from RCL-IIOS (2001–2014)	[69]	High: >0.2 mg/l; Low: ≤ 0.2 mg/l
	Chlo-a epilimnion	Child	Data	RCL-IIOS (2001–2014), [10]	[74]	High: ≥3 mg/l; Low: 0–3 mg/l
	Respiration rate epilimnion	Child	Expert knowledge	Based on one-time data measurement from RCL-IIOS	Expert knowledge recalibrated with sensitivity to parameter (to DO epilimnion)	High; Low
	Respiration rate metalimnion	Child	Expert knowledge	Based on one-time data measurement from RCL-IIOS	Expert knowledge	High; Low
	BOD metalimnion	Child	Expert knowledge	Based on [72, 73, 75]]	[69]	High: ≥3 mg/l; Low: <3 mg/l
	PO4 Released from hypolimnion	Child	Expert knowledge	Based on [10, 76]	[10]	High; Low
	Reactive Fe (Fe^2+^) Concentration	Parent	Expert knowledge	Based on [10]	[10]	Low
	Chlo-a metalimnion	Child	Expert knowledge	Based on [10]	[74]	High: ≥3 mg/l; Low: 0–3 mg/l
	H_2_S	Child	Data	[77]		High:>0.002 mg/l; Low: ≤ 0.002 mg/l
	GPP epilimnion	Child	Expert knowledge	Based on one-time data measurement from RCL-IIOS	Expert knowledge recalibrated with sensitivity to parameter (to Anoxic layer)	High; Low
	GPP metalimnion	Child	Expert knowledge	Based on one-time data measurement from RCL-IIOS	Expert knowledge recalibrated with sensitivity to parameter (to Anoxic layer)	High; Low
	DO epilimnion	Child	Data	RCL-IIOS (2005–2018)	[70]	High:>3.5–6 mg/l; Low ≤3.5mg/l
	DO metalimnion	Child	Data	RCL-IIOS (2005–2018)	[70]	High:> 3.5–6 mg/l; Low ≤3.5mg/l
Human dimension (input nodes)	Stocking density	Child	Expert knowledge	Interview with IWCC farmers and staffs of Fisheries Agency	[73]	Low: ≤ 200 seeds/m^2^; Medium: 200–500 seeds/ m^2^; high: 500–800 seeds/ m^2^
	Number of active cages	Child	Expert knowledge	Interview with staffs of Fisheries Agency and data from Fisheries Agency	Expert knowledge	Zero; Low: <2,000 units; Medium: <2,000–6,000 units; High: ≥6,000 units
	Feeding management	Parent	Expert knowledge	Interview with IWCC farmers and [73]	[73]	None; Once-Floating; Twice-Floating; Once-Emerge; Twice-Emerge
	Organic sediment run off	Child	Expert knowledge	Validated with [72, 78]	[72]	Low:0–60 tonnes/ha/year; Medium:61–180 tonnes/ha/year; High: >180 tonnes/ha/year
	Feed	Child	Expert knowledge	Based on [73] evaluated with interview results with IWCC farmers	Expert knowledge	High: ≥2500 kg/unit/farming cycle, Medium:1,500–2,500 kg/unit/farming cycle, Low:500–1,500 kg/unit/farming cycle
	Accumulated fish feed	Child	Expert knowledge	Based on [75]	Expert knowledge	High; Low
Output nodes	*Gobiopterus* disappearance	Child	-		-	-
	Mass fish kills	Child	-		-	-

A mix of data and expert domains were engaged to fill Conditional Probability Tables (CPT) referring to the work of [64]. The data to set prior probabilities were obtained from various sources (Table 1). Most ecological data (i.e., DO, Chlo-a) were obtained from measurement data of the Research Centre for Limnology-Indonesian Institute of Sciences (RCL-IIOS). Meanwhile, most daily climatic data (rainfall intensity, wind speed, wind direction, cloudy) were collected from Meteorology, Climatology, and Geophysical Agency (BMKG). The data presented in high-frequency measurement data (measured every 2 to 10 minutes) were processed into the most frequent occurrence data (i.e., wind direction, wind speed) and maximum value data (rainfall intensity).

Other CPTs (i.e., H_2_S, DO-metalimnion, phosphate released from sediment) were set by using expert knowledge referring to peer-reviewed publications and reports from Government Institutions. They were calibrated after conducting repeated sensitivity to parameters tests (to mixing, MFK, and *Gobiopterus* disappearance). Schmidt Stability Index (SSI), which shows the lake’s resistance to mechanical mixing [68] was calculated by using R-lake analyser from high-frequency measurement data from RCL-IIOS (2014–2017). The trend of SSI related to light intensity was used as the basis to fill the CPT. Detail of the method used to fill CPT of each node is presented in Table 1 and S3 Table in S1 File.

### Model evaluation

#### Sensitivity to findings

Sensitivity to findings helps to identify errors in either the network or the CPTs [46, 64]. First, the analysis was conducted to MFK and *Gobiopterus* disappearance node. The analysis results show that mixing and epilimnion zone after mixing nodes are the most influencing factors for both output modes. These results are consistent with the model as these two nodes are the direct nodes connected to the output nodes. Next, the sensitivity to findings was applied to find the most influenced factors to mixing and epilimnion zone after mixing. The sensitivity to findings is illustrated in Mutual Information Value. Mutual Information Value is the subtraction of entropy/ H(X) and the probability of distribution of the entropy given prior condition (H (X│Y)). This value is used to rank the most influencing factors to the output nodes, please refer to [64] for details.

#### Model accuracy evaluation

A goodness of fit procedure using past field observation data of MFK events was used to measure model accuracy [46, 60, 64]. Three indicators used to show the model accuracy is the error rate, true skill statistics, and Area Under Curve (AUC). Unfortunately, there is no record data of the abundance of *Gobiopterus*; thus, model accuracy evaluation cannot be performed to ‘*Gobiopterus* disappearance’ node.

The past observation data include wind speed, wind direction data, and the dates when MFK events happened. The data were obtained from mixed data from RCL-IIOS online monitoring device and observation data from Agam Fisheries Agency. The data from the RCL-IIOS were recorded in a high-frequency observation from mid of July to early August 2019, December 2019, and January-February 2020 (n = 8,331 data sets). These data were then processed into frequent wind speed and wind direction data resulting in 100 observation points. Meanwhile, among 20 MFK event dates only nine dates can be used, because there were no climatological data for the other 11 dates. The data is then inputted to the model to obtain the prediction of MFK probabilities, coded as predicted MFK. Meanwhile, the actual MFK events were coded as observed MFK.

The predicted MFK probability was coded as Yes = 1 or No = 0 using various cut-off points (1% to 96%). Then the predicted probability distribution is compared with actual MFK event occurrence to calculate the sensitivity and specificity of the model. The obtained sensitivity and 1-specificity were plotted to form a receiving operating curve (ROC) (see [67, 79]). A non-parametric ROC was generated by using Stata 15 software following the methodology proposed by [80]. A Non-parametric ROC curve was selected as it does not require assumptions of a normal distribution of specificity and sensitivity [81]. The area under the ROC curve (AUC) is used as the indicator of model accuracy.

The next step is the calculation of an error rate following [65]. First, an optimum cut-off point should be obtained to code the projected probabilities into MFK predicted occurrence (Yes = 1 and No = 0). The optimum cut-off point was determined by engaging the minimum P-value approach [82, 83]. The optimum cut-off value is the cut point resulting in maximum χ^2^ value. Then, using this cut-off point, a 2x2 cross table of predicted MFK and observed MFK was produced. Finally, the error rate is calculated by dividing the incorrect prediction with total observation (n = 100). The true skill statistic (TSS) was also calculated at the obtained cut-off point. This value of TSS was obtained using the equation suggested in [84, 85]: TSS = Sensitivity +specifity-1.

### Management scenarios

Several management scenarios are presented. These scenarios are grouped as short-term (immediate response) and longer-term (up to 25 years) management actions. The short-term management options include with and without complete removal of IWCCF to test the implications of complete removal of IWCCF such as suggested by the environmentalists, reduction IWCCF of up to 6,000 cages (proposed by the Act of Bupati Agam No. 22 /2009), and reduction of internal phosphorous loading (IPL) (i.e., by sediment capping) and aeration recommended by aquaculture practitioners. All the short-term scenarios are generated in the model setting representing the current lake condition (i.e., high H_2_S and high IPL).

For longer-term management options, a continuation of current IWCFF situation and present lake condition as the result of no management action, complete removal of IWCCF, and reduction IWCCF up to 6,000 will be the proposed actions. A timeframe of maximum 25 years was chosen as this corresponds to the water retention time of Lake Maninjau [86]. The best management scenarios are chosen based on their ability to reduce the probabilities of MFK and *Gobiopterus* disappearance, while also considering social implications of the scenarios.

## Results

### Constructed BBN

**Fig 2:**
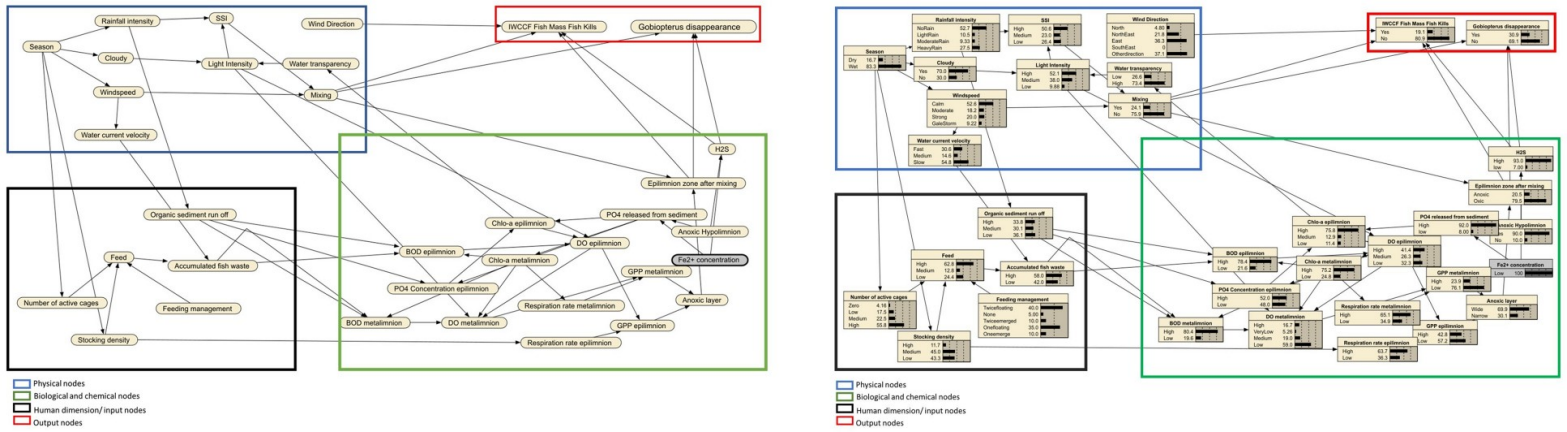
The constructed BBN model. (A) The conceptual model (B) The belief bar.

The built labelled box style of the model comprises physical, chemical, biological, and human dimension (input) nodes related to IWCCF and lake ecosystem is shown in Fig 2A. An alternative form of model presentation, the belief bar style (the model completed with the CPT tables), is shown in Fig 2B. The first three nodes are regarded as intermediate nodes, and the human dimension is further referred to as input nodes. Details of the variables can be accessed in Table 1. Interconnections of these parameters result in two targeted outputs (IWCCF Fish MFK and *Gobiopterus* disappearance). These two outputs represent the criteria of interest for informing decision-makers about trade-offs between IWCCF and the abundance of *Gobiopterus*.

### Results of sensitivity to findings

**Fig 3:**
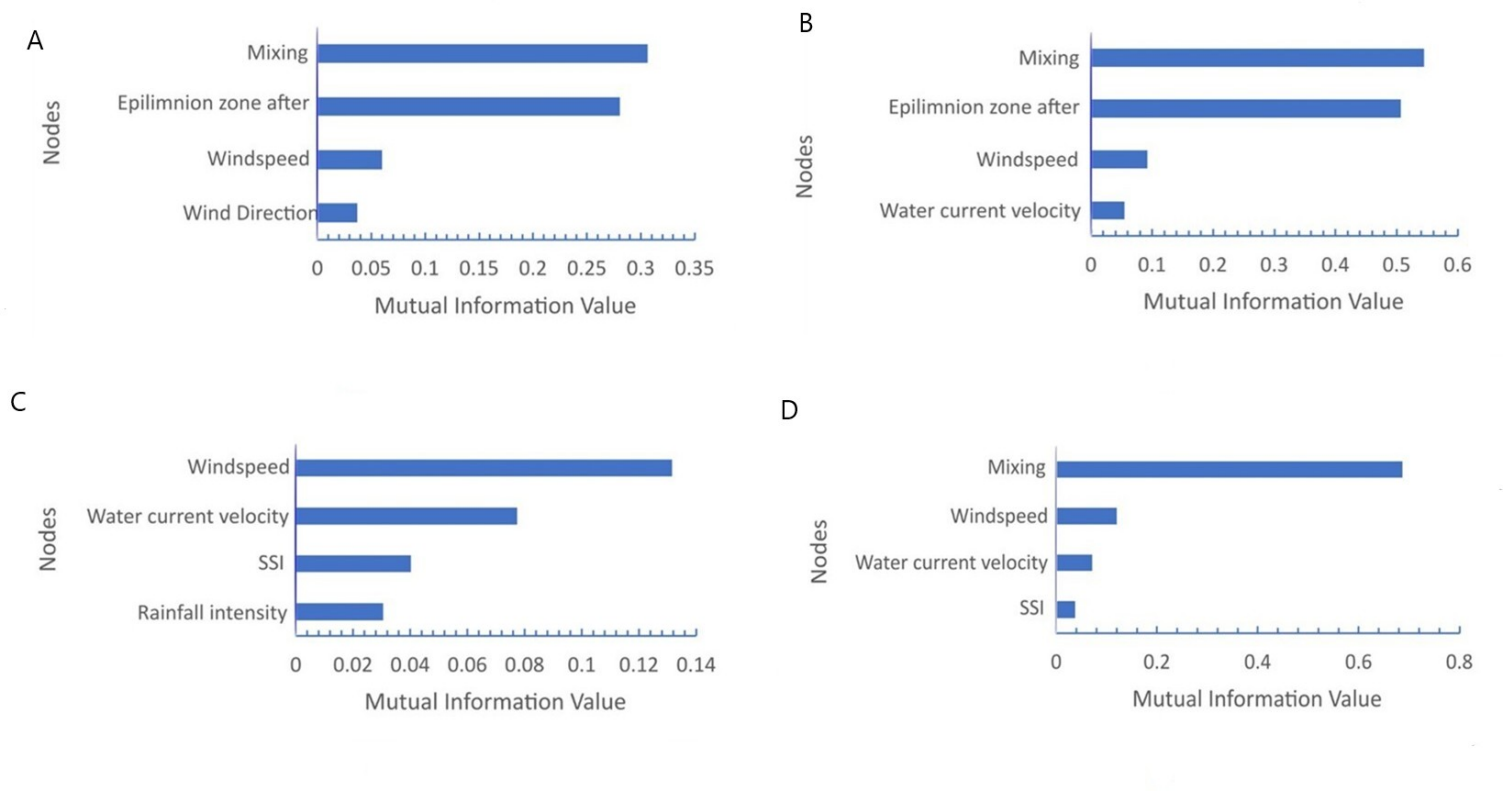
Sensitivity to findings (presented in Mutual Information Value) of four most influential parameters to A. MFK; B. *Gobiopterus* disappearance; C. Mixing; D. Epilimnion zone after mixing.

Fig 3 presents the sensitivity to findings (presented as Mutual Information Value) of four most influential parameters to changes in the nodes “mixing” and “epilimnion zone after mixing”. The Figure also shows the results of sensitivity to findings to the output nodes. As it has been explained in Sensitivity to Findings Section, “mixing” and “epilimnion zone after mixing” nodes are the most influential factors of both output nodes. Thus, these two intermediate nodes reflect the influence of other parameters towards the output nodes. In the Figure, the horizontal axis shows the mutual information value, which is a measurement of influence of one node on other nodes [64]. The sensitivity to findings of changes in other factors are not presented here due to space limitations.

It can be seen in the Figure that wind speed, water current velocity, SSI, and rainfall intensity are the most affecting parameters. Thus, it can be seen that physical factors especially wind speed, are the principal drivers of MFK and *Gobiopterus* disappearance events. Further, it is also suggested that the output nodes are insensitive to the current lake condition, controlling external phosphate loading by regulating the number of cages, stocking density, and feed management (input nodes). Please refer to discussion section to read the detail explanation.

### Results of model accuracy evaluation

An optimum cut-off value is presented to balance between the sensitivity (true positive) and specificity (true negative) results. An optimum cut-off value of 26% was calculated (Table 2). A high cut-off value (e.g. 50%) will increase the possibility of true negative results but will reduce the possibility of true positive results. On the contrary, if the cut-off point is too low (e.g. 5%), it will increase true positive results but reduce the true negative results. Imbalance between sensitivity and specificity will eventually raise the model error rates.

**Table 2:** Sensitivity, specificity, calculated χ^2^, and P value of several cut-off points.

Cut-off point (%)	Sensitivity	Specificity	Calculated χ^2^	P-value
0	1.000	0.000	-	-
1	0.157	1.000	5.297	0.021
4	0.155	1.000	5.048	0.025
7	0.159	1.000	5.553	0.018
10	0.375	0.974	22.652	0.000
13	0.440	1.000	37.079	0.000
15	0.450	0.975	29.520	0.000
18	0.474	0.975	31.691	0.000
20	0.429	0.975	27.556	0.000
23	0.474	0.975	31.691	0.000
26*	0.818	0.978	63.315	0.000
30	0.818	0.978	63.315	0.000
50	0.818	0.978	63.315	0.000

**Fig 4:**
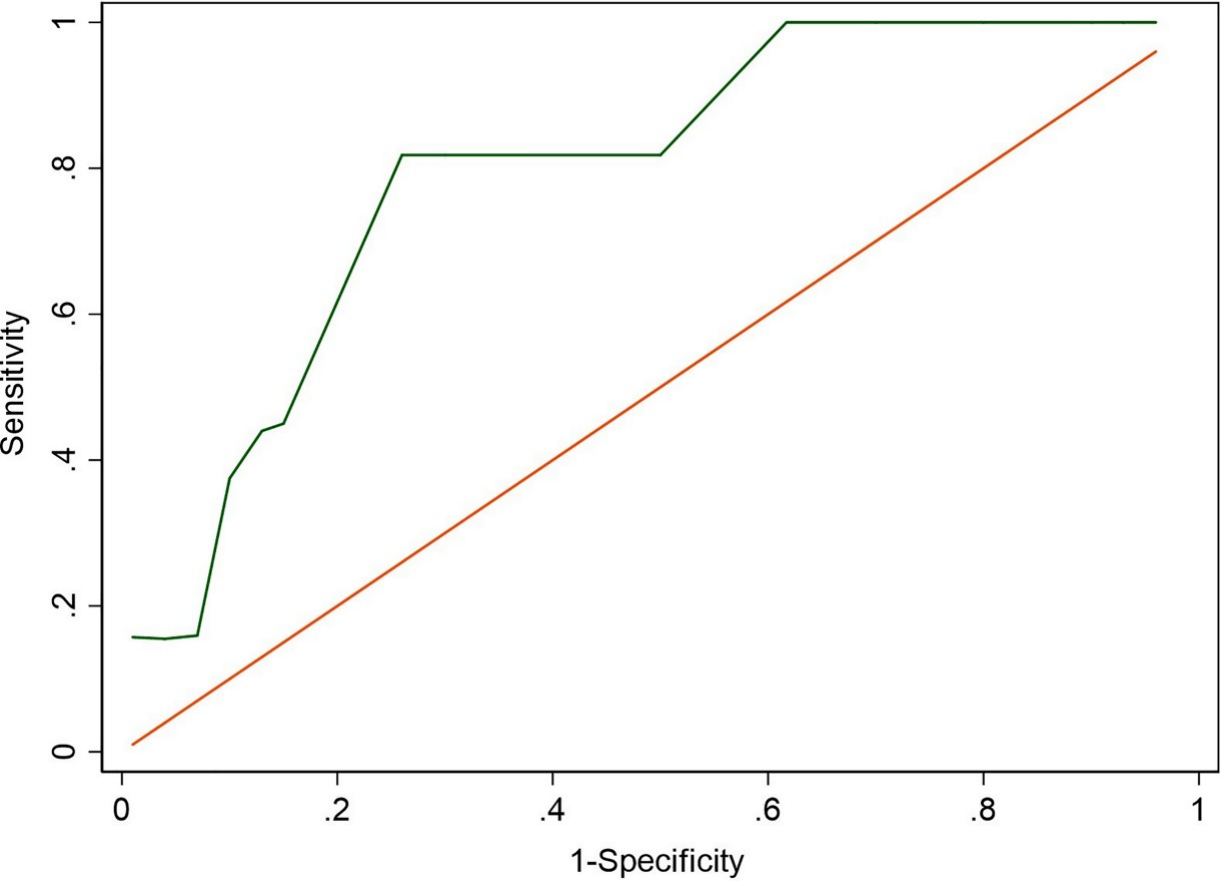
Non-parametric Receiving Operating Curve (ROC). Area under ROC: 0.897.

The non-parametric ROC is presented in Fig 4, showing the AUC is 0.9. The AUC value shows that the constructed BBN model is useful [87] with moderate to excellent discrimination [79, 88]. The computed error of the model at the optimum cut-off point is 4% consisting of four wrong predictions among 100 tested data points (Table 3). The information in the table shows that there were two predicted MFKs that were not observed in actual observations. At the same time, there were also two MFK events that were not predicted by the model. This result is relatively low compared to other BBN models (i.e., 9.62% for wildfire occurrence [67]). However, the categorization of high and low error rate is somewhat different for each subject of study.

**Table 3:** Confusion matrix of observed and predicted MFK at cut-point 26%.

Observed MFK	Predicted MFK		
		No	Yes
	No	Specificity = 87	False positive = 2
	Yes	False Negative = 2	Sensitivity = 9
N = 100			

The calculated TSS is 0.796. The TSS value has a range from -1 to 1, with the value below zero indicating that model performance is based on chance alone (random) [84, 85]. This means that the model can produce strong prediction of MFK with the available data and the correct prediction are not based on random incidence; thus, it can be considered to be statistically robust.

### Results of predicted probability of MFK and *Gobiopterus* disappearance for several management scenarios

**Fig 5:**
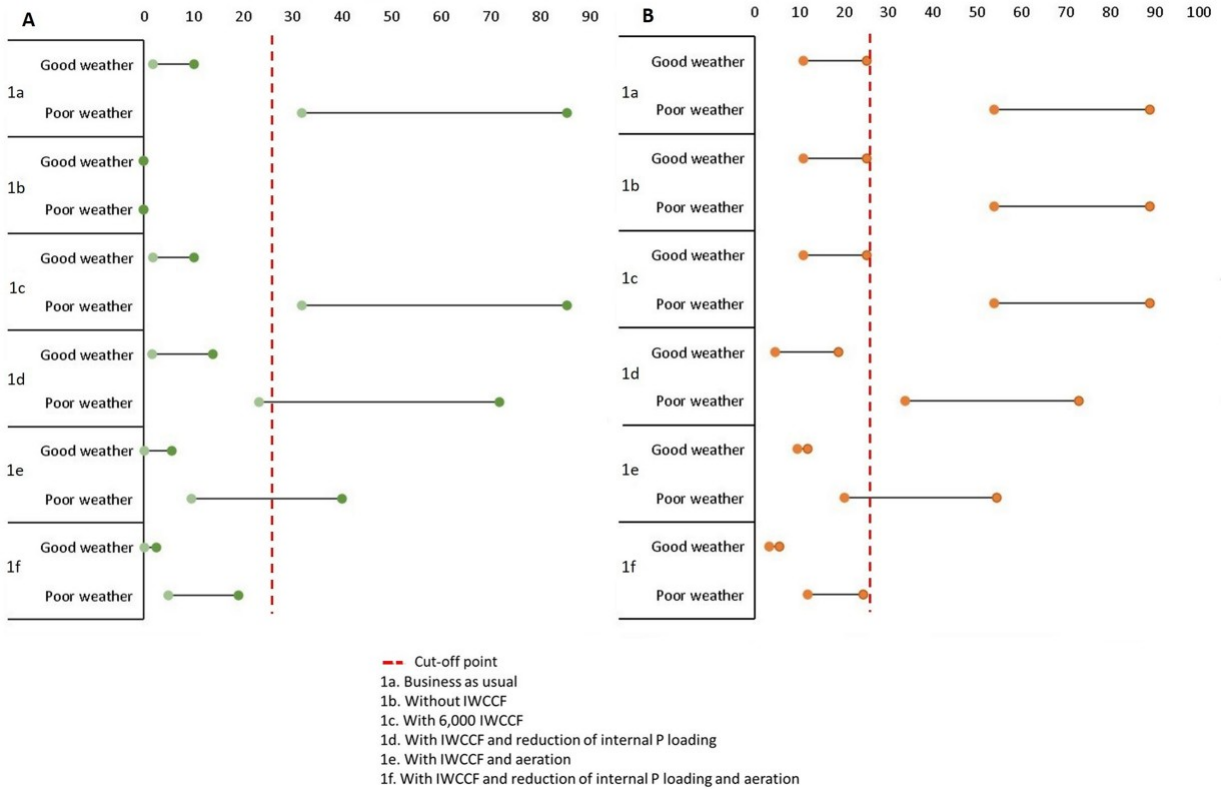
Predicted probability of several short-term management scenarios representing current condition. (A) MFK, (B) *Gobiopterus* disappearance.

**Fig 6:**
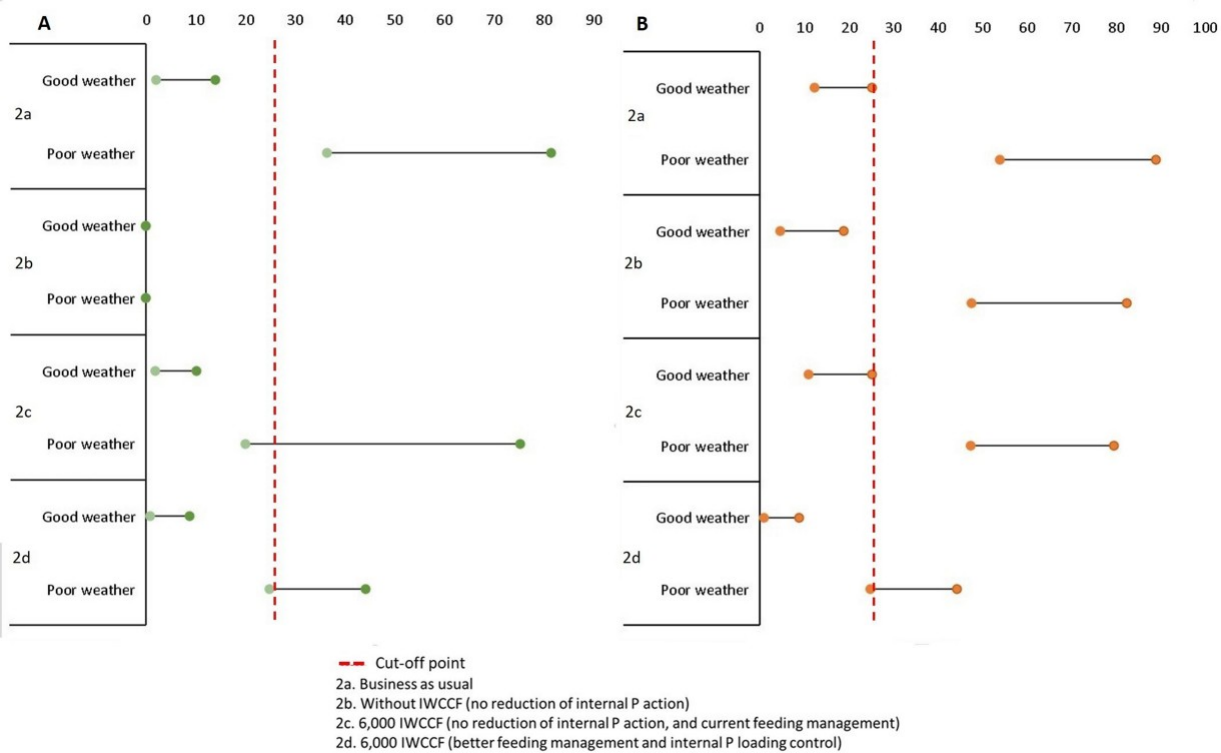
Predicted probability of several longer-term management scenarios representing current condition. (A) MFK, (B) *Gobiopterus* disappearance.

Figs 5 and 6 present the predicted probability of MFK and *Gobiopterus* disappearance with several management scenarios. The summary of the data presented in those figures is shown in Table 4, and the details can be seen in S4 Table in S1 File. The scenarios are categorized as the short-term impacts of the available management options (Scenario 1a–1f) and the longer-term effects (Scenario 2a–2d). The value in the table shows predicted probability which is translated to yes (the events happen) and no (the events do not happen) by using the calculated optimum cut-off value (see Model Accuracy Evaluation Section). As an example: for Scenario 1a, the predicted probability of MFK in good weather condition is 1.8–17%; this means that no MFK event is predicted as the value is lower than 26%. At the same time, in bad weather condition, the predicted probability is 32–85.5%, which means it is highly likely that MFK will occur.

**Table 4:** Predicted probability of MFK and *Gobiopterus* disappearance with several management scenarios.

Condition	Management scenarios		Expected Value of Predicted probability (%)			
			Cultured fish MFK		*Gobiopterus* disappearance	
			Dry season (calm wind, No rain-moderate rain)	Wet season (Strong-gale-storm wind, moderate-heavy rain)	Dry season (calm wind, No rain-moderate rain)	Wet season (Strong-gale-storm wind, moderate-heavy rain)
Current condition (high H_2_S, high internal P release from sediment)	1a	Business as usual	NO (1.77–10.1)	YES (32–85.5)	NO (10.9–25.2)	YES (54.0–89.0)
	1b	Without IWCCF	NO 0^a^	NO 0^a^	NO (10.9–25.2)	YES (54.0–89.0)
	1c	With 6,000 IWCCF	NO (1.77–10.1)	YES (32–85.5)	NO (10.9–25.2)	YES (54.0–89.0)
	1d	With IWCCF and reduction of internal P loading	NO (1.6–13.9)	YES (23.3–71.9)	NO (4.56–18.8)	YES (33.8–72.9)
	1e	With IWCCF and aeration	NO (0.21–5.67)	NO/YES (9.6–40.1)	NO (9.53–11.9)	NO/YES (20.1–54.5)
	1f	With IWCCF and reduction of internal P loading and aeration	NO (0.42–2.52)	NO (4.96–19.1)	NO (3.23–5.61)	NO (11.9–24.4)
Longer-term (up to 25 years)	2a	Business as usual	NO (2.02–14)	YES (36.5–81.6)	NO (12.2–25.3)	YES 54–89.0
	2b^b^	Without IWCCF (no reduction of internal P action)	NO 0^a^	NO 0^a^	NO (4.56–18.9)	YES (47.5–82.5)
	2c^b^	6,000 IWCCF (no reduction of internal P action, and current feeding management)	NO (1.77–10.1)	YES (20–75.3)	NO (10.9–25.2)	YES (47.4–79.5)
	2d	6,000 IWCCF (better feeding management and internal P loading control)	NO (0.85–8.83)	NO/YES (24.8–44.2)	NO (0.85–8.83)	NO/YES (24.8–44.2)

Note:

^a^ No IWCCF in the scenario.

^b^ see ‘Results of predicted probability of MFK and *Gobiopterus* disappearance for several management scenarios’ Section for the assumptions.

The predicted probability is shown in two contrasting weather conditions. The first one (good weather condition characterized by dry season, calm wind, no heavy rain) represents the optimum condition when MFK and *Gobiopterus* disappearance is the least likely to occur. On the contrary, the second condition (poor weather condition associated with wet season, gale-storm wind, heavy rain) represents the highest possibility of MFK and *Gobiopterus* disappearance to happen.

The short-term scenarios reflect the expected probabilities of occurrence of both MFK events and *Gobiopterus* disappearance within the near future after the chosen management actions are imposed. The management options can be seen in the management Scenarios Section. The longer-term effect scenarios project the impacts of the management to the expected probabilities of both output nodes up to 25 years. This time frame is chosen because it is the water retention time of the lake water [86].

We set the ‘PO_4_ released from sediment’ and ‘Anoxic layer’ to the lower state to represent the expected condition in longer term after the reduction and eradication of the cages making the fulfilment of the assumption become vital.

There are two assumptions used to run scenario 2b (longer-term effects of complete removal of IWCCF) and scenario 2c (longer-term effects of keeping 6,000 IWCCF). The first assumption is that there are no other factors inhibiting lake self-purification (i.e., continuing IPL and absence of stable macrophytes communities) (see [89, 90]). The second assumption is that the rate of external organic sediment run-off (i.e., domestic waste) is unchanged.

The results show that in the short-term, removing IWCCF will not have a significant impact on *Gobiopterus* disappearance, although the losses due to MFK can be mitigated (Scenario 1a vs 1b). Meanwhile, keeping 6,000 IWCCF offers similar results as the business-as-usual scenario (Scenario 1a vs 1c). More importantly, combating the IPL is expected to improve the lake condition and may reduce the probability of MFK and *Gobiopterus* disappearance considerably (Scenario 1a vs 1d). Overall, the suggested best short-term management option is by conducting aeration alone or combine it with a reduction of IPL (Scenario 1e and 1f).

For the longer-term scenarios, removing all IWCCF may become the best option to avoid MFK events and *Gobiopterus* disappearance as long as the assumptions above are fulfilled (Scenario 2b). Further, limiting the number of IWCCF to a moderate amount (e.g. 6,000) may cut probability of MFK and *Gobiopterus* disappearance by more than half, if it is complemented with a reduction of IPL and better feeding management (Scenario 2a vs 2d). Without performing these efforts, a less significant improvement can result from reducing the cages to the 6,000 units (Scenario 2a vs 2c).

## Discussion

The constructed BBN model can provide a relatively accurate prediction of MFK when tested with only two explanatory variables (wind speed and wind direction). This finding indicates that the developed model can perform well with limited data availability. The BBN’s ability to incorporate both data learning and expert knowledge is the principal benefit in supporting this feature. The model, therefore, can be useful to assist decision-making in the study area.

Further, the model results show that weather conditions, especially wind speed, can be used as a good predictor of the outputs. Therefore, in the current lake condition, forecasting wind speed is crucial to minimize financial loss during MFK by harvesting the fish from cages before stormy events. However, some loss is inevitable in such a strategy, because not all fish will have reached the most profitable size. Interestingly, some local IWCCF farmers have adopted this adaptation strategy in recent years [44]. They harvest the farmed fish one or two days before strong wind is predicted by the weather forecast, and they reduce stocking rates and production during the rainy season.

Our previous study [44] has correlated the farmers’ strategy with the prediction of more frequently extreme weather such as *La Niña* which likely triggers more upwelling in the study area. Thus, it gives us an insight that developing the model to take into account the climate change impacts in the future is necessary aligning with the work of [91].

In the developed model, the output nodes are not sensitive to the input nodes/human dimension nodes. In BBN, the sensitivity of output nodes towards input nodes can be reduced by a large number of intermediate nodes [23, 65]. Thus, [65] advised to limit the number of nodes to not exceed 40. The model uses 35 nodes in line with their advice. This would suggest that model over-complexity is unlikely to be the cause of the lack of output nodes sensitivity to the input nodes. One possible explanation is that high rates of IPL and H_2_S mimic the current condition of the lake (see Materials and Methods Section). This shows that even if the IWCCF was eradicated, it will not immediately result in water quality improvement. In line with this finding, [89, 90, 92] elucidated that chemical and biological resistance factors (i.e., IPL) can delay water quality improvement in shallow lakes. IPL can endure for 10–15 years after the external load is reduced [93, 94]. However, [95] mentioned that deep stratified lakes typically have shorter longevity of internal sediment P released than the shallow lakes. Thus, limiting IPL is one of the main actions that should be conducted to achieve desired results in terms of reduced MFK frequency and presence of *Gobiopterus*. Further, it shows that longer-term result of complete removal of IWCCF will be achieved if other factors inhibiting lake self-purification such as IPL is controlled.

Thus, the necessity to recognize IPL as one of the primary causes of water quality deterioration and the needs of restoration actions to combat it becomes the main lesson learnt from the application of the model. International literature recognizing the importance of acknowledging IPL and the significance to combat it in the deep tropical lakes is scarce. Until recently, IPL control is not included in the priority list in most deep lake restoration programmes in tropical countries (See [44, 96–98] for literature on national restoration plans of several lakes in India, Thailand, and Indonesia). At the same time, most related research are case studies in shallow temperate lakes (e.g., [95, 99–101]) and in shallow subtropical and tropical lakes (e.g., [102, 103]).

In the short-term, conducting aeration may significantly reduce the probability of MFK and *Gobiopterus* disappearance (Scenario 1e). This action commonly practiced in pond aquaculture by infusing oxygen in the water column is aimed at reducing the probability of fish death (see [104, 105]). In 2019, research related to the application of nano-bubble technology epilimnion aeration was conducted in Lake Maninjau. The research was done by a collaborative research team of RCL-IIOS and The Ministry of Environment and Forestry (see [44]). Further, combining epilimnion aeration and other efforts to control IPL is considered as the best option to reduce the probability of MFK and *Gobiopterus* disappearance events (Scenario 1f). The aeration can be placed in the IWCCF area. In the future, development of spatial BBN is necessary to advise which areas should be prioritized to be aerated.

The significance of spatial BBN has been demonstrated by [106–108]. They use the spatial model to map the prioritize management intervention area, to incorporate stakeholders’ perspectives, and to map landscape vulnerability and concluded that spatial BBN is a practical tool to achieve their objectives.

Although total cage removal can make the possibility of MFK and *Gobiopterus* disappearance more unlikely in the longer-term, other considerations such as poverty alleviation and significant potential to create social conflict (see [15, 42]) should also be thoroughly assessed. It was reported that conflicts of interest between water users developed into riots in 2001 [103]. Thus, a balance between ecology and economy should be achieved considering these circumstances. This is particularly important if the impacts of actions such as cage removal are not certain to alleviate MFK. One alternative that can be proposed from this model is the integration of efforts to reduce IPL while maintaining a moderate amount of IWCCF (Scenario 2d). Various alternatives of techniques to reduce IPL are available. Two promising methods that can be applied are sediment interface aeration, and sediment capping [109–111].

To provide a more comprehensive assessment of both ecology and economy impacts of the scenarios, an endpoint such as a utility node defined in terms of effects on income/livelihood from IWCCF can be a useful amendment to the current model. Towards this end, deriving high quality time series data about the economic aspects of IWCCF and local residents’ livelihood will be of high priority for future research. This would allow a more detailed quantification of trade-offs between IWCCF and native fisheries in order to inform management decisions [23]. There is a clear necessity of quantifying ES trade-offs using specific modelling tools such as BBN [27]. In the study area, information of quantification of ES trade-off is very useful to help the decision makers to allocate their limited financial budget on relatively more sustainable management actions. However, monetary valuation of trade-offs between IWCCF and native fisheries is beyond the scope of this paper.

The main limitation of the developed BBN model is the difficulty to integrate dynamic observations to provide the exact point in time when the IPL starts taking place. In this case, mechanistic models are required to complement the BBN model. Unfortunately, for the time being, such models are not available yet in the study area, and their development may be challenging and time consuming due to the complexity of this tropical lake ecosystem. Some studies have demonstrated the use of BBN to complement other models [58, 112–114]. These studies highlight that BBN generate results that were more user-friendly and more easily understood by end users and decision makers; nevertheless, mechanistic models can provide more detail and thus also help refining inputs for the BBN.

In this case study, the BBN points to a need for an improved understanding information gaps which is applicable to the lake management in Indonesia and the Global South in general. In short, the information gaps found by application of the constructed model are:

1. Absence of high-quality time series data on the limnological aspects of the lake to create a mechanistic model to complement the built BBN model as the built BBN model has several aforementioned limitations.
2. Lack of observational time series data on the impacts of phosphate reduction on water quality improvement which leads to poor understanding of the underlying mechanism that may delay the effects of phosphorous reduction in tropical lakes.
3. Lack of high-quality time series data about the economic aspects of IWCCF and local residents’ livelihood.
4. Need to conduct an economic valuation of the management scenarios to assists the decision-making process.

Other studies aligning with these findings are for example [96] voicing the importance to conduct economic valuation research to support lake management in India. Further, [115] identified that inadequacy of basic limnological monitoring data hindered effective science-based policy lake management in the Philippines.

## Conclusion

The constructed BBN model can be used to capture the complexity of the ecological system in a deep tropical lake. It was constructed by using both data and expert and stakeholder knowledge approaches. However, careful model parameterisation and validation is required when engaging in this approach. It overcomes limited data availability to overcome limitations regarding data availability that often characterise such systems and is shown to be accurate when tested with past field data observations (an error rate of 4%, TSS of 0.8, and AUC of 0.9).

The model can assist decision making by highlighting the importance of wind speed forecasts to reduce the loss of MFK. Other important factors that should be observed are related to lake stability (i.e., SSI and rainfall intensity). Further, it reflects that reducing external phosphorus loading (in this case by reducing the number of cages used for fish farming on the lake) may not give expected results within a short time and also in the longer-term, if IPL is not controlled. Thus, the findings underline the necessity of actions to combat IPL in deep tropical lake systems.

In the short-term, combining control of internal phosphate released with aeration is considered the best option to reduce the probabilities of MFK and *Gobiopterus* disappearance. In the longer-term, maintaining a moderate amount of the cages along with efforts to control IPL may become an alternative solution to balance the lake’s ecological condition and to conserve economic benefits. Together, this may also help alleviating social conflicts in the study area and elsewhere.

The use of BBN does not undermine the importance of availability of adequate data to create a mechanistic model. Rather, it should be complemented by such model to provide more comprehensive decision making. Further, the identified data and knowledge gaps are relevant to improving lake management in the tropical system in general.

## Supporting information

S1 File (DOCX)
