# Supplementary material for: An application of Bayesian Belief Networks to assess management scenarios for aquaculture in a complex tropical lake system in Indonesia
Source: PLoS One. 2021 Apr 16;16(4):e0250365. doi: 10.1371/journal.pone.0250365 (PMC8051808; doi:10.1371/journal.pone.0250365)
Supplement: S1 File — (DOCX) [file pone.0250365.s001.docx]

# S1 Table. Line-up of literature used to conceptualise the BBN model

| Model nodes | Literature(s) |
| --- | --- |
| Season -Rainfall intensity | [1] |
| Season-cloudy days | [1] |
| Season-Wind speed | BMKG |
| Season-Number of active cages | Interview |
| Rainfall intensity-SSI | [2,3] |
| Rainfall intensity-Organic sediment run-off | (Ministry of Public Work. 2013) |
| Cloudy days-Light intensity |  |
| Light intensity-SSI | [5] |
| Light intensity-DO epilimnion | [6] |
| Light intensity-DO metalimnion | [6,7] |
| SSI-Mixing | [3] |
| Water transparency-Light intensity | [8] |
| Wind speed-Water current velocity | [9,10] |
| Wind speed-Mixing | [11] |
| Wind direction-Mixing | [4,11] |
| Water current velocity-Accumulated feed waste | [5] |
| Mixing-Epilimnion zone after mixing | [12] |
| Mixing-Mass fish kills | [13,14] |
| Mixing-*Gobiopterus* disappearance | Interview |
| Epilimnion zone after mixing- Mass fish kills | [14] |
| Epilimnion zone after mixing- *Gobiopterus* disappearance | Interview |
| Anoxic layer- Epilimnion zone after mixing | [5,15] |
| DO epilimnion-DO metalimnion | [16] |
| DO epilimnion-GPP epilimnion | [17] |
| DO metalimnetic-GPP metalimnion | [17] |
| H_2_S-Mass fish kills | [18–20] |
| H_2_S -*Gobiopterus* disappearance | [18], interview |
| Fe concentration- H_2_S | [20–22] |
| Fe concentration- PO4 Released from sediment | [20,22,23] |
| Anoxic hypolimnion- PO4 Released from sediment | [22,24,25] |
| Anoxic hypolimnion- H_2_S | [24] |
| PO4 Released from sediment- PO4 concentration epilimnion | [20] |
| PO4 Released from sediment- Chlo-a epilimnion | [20] |
| PO4 Released from sediment- Chlo-a metalimnion | [20] |
| BOD epilimnion-DO epilimnion | [26] |
| BOD metalimnion-DO metalimnion | [26] |
| PO4 concentration epilimnion- Chlo-a epilimnion | [27,28] |
| GPP epilimnion-Anoxic layer | [29] |
| GPP metalimnion-Anoxic layer | [29] |
| Chlo-a epilimnion-Water transparency | [15] |
| Chlo-a epilimnion-DO epilimnion | [30,31] |
| Chlo-a metalimnion-DO metalimnion | [30,31] |
| Chlo-a metalimnion-BOD epilimnion | [32,33] |
| Chlo-a metalimnion-BOD metalimnion | [32,33] |
| Chlo-a metalimnion-Respiration rate metalimnion | [34,35] |
| Respiration rate epilimnion-GPP epilmnion | [29,36] |
| Respiration rate metalimnion-GPP metalimnion | [29,36] |
| Accumulated feed waste-BOD epilimnion | [37] |
| Accumulated feed waste-BOD metalimnion | [37] |
| Organic sediment run off-PO4 concentration epilimnion | [4,38] |
| Organic sediment run off-BOD epilimnion | (Ministry of Public Work 2013) |
| Organic sediment run off-BOD metalimnion | [4,38] |
| Feeding management-feed | [39] |
| Stocking density-Respiration rate epilimnion | [40] |
| Stocking density-Feed | [39] |
| Number of active cages-feed | Interview |
| Feed-Accumulated feed waste | [41] |

References

1. Ulfah A, Sulistya W. Penentuan Kriteria Musim Alternatif Di Wilayah Jawa Timur. J Meteorol dan Geofis. 2015;16: 145–153.

2. Rooney GG, Lipzig N Van, Thiery W. Estimating the effect of rainfall on the surface temperature of a tropical lake. Hydrol earth Syst Sci. 2018;22: 6357–6369.

3. Read JS, Hamilton DP, Jones ID, Muraoka K, Winslow LA, Kroiss R, et al. Derivation of lake mixing and stratification indices from high-resolution lake buoy data. Environ Model Softw. 2011;26: 1325–1336. doi:10.1016/j.envsoft.2011.05.006

4. Ministry of Public Work. Laporan Akhir Pekerjaan: Studi konservasi kawasan Danau Maninjau di Kabupaten Agam Provinsi Sumatera Barat. 2013. doi:10.1017/CBO9781107415324.004

5. Santoso AB, Triwisesa E, Fakhrudin M, Harsono E, Rustini HA. What do we know about Indonesian tropical lakes ? Insights from high frequency measurement What do we know about Indonesian tropical lakes ? Insights from high frequency measurement. IOP Conf Ser: Earth Environ Sci 118. 2018. pp. 1–5. doi:118 012024

6. Eilers PHC, Peeters JCH. A model for the relationship between light intensity and the rate of photosynthesis in phytoplankton. Ecol Modell. 1988;42: 199–215.

7. Adams MS, Titus J, Mccracken M. Depth distribution of photosynthetic activity in a Myriophyllum spicatum community in Lake Wingral. Limnol Oceanogr. 1974;19.

8. Clarke GL. Light as a limiting factor for aquatic animals and plants. Am Biol Teach. 1939;1: 150–154.

9. Henderson-Sellers B. The dependence of surface velocity in water bodies on wind velocity and latitude. Appl Math Model. 1988;12: 202–203.

10. Haines DA, Bryson RA. An empirical study of wind factor in Lake Mendota. Limnol Oceanogr. 1961;6: 356–364.

11. Magee MR, Wu CH. Response of water temperatures and stratification to changing climate in three lakes with different morphometry. Hydrol earth Syst Sci. 2017;21: 6253–6274.

12. Schmid Ma, Alfred Wuest. Stratification, mixing, and transport processes in Lake Kivu. In: Descy J-P, Schmid M, Darchambeau F, editors. Lake Kivu Limnology and Biogeochemistry of a tropical great lake. New York: Springer Dordrecht Heidelberg; 2012. pp. 13–19.

13. Sulastri, Sulawesty F, Nomosatryo S. Long term monitoring of water quality and phytoplankton changes in Lake Maninjau, West Sumatra, Indonesia. Oseanologi dan Limnol di Indones. 2015;41: 339–353. Available: file:///J:/Data/Computer/Config/Citavi5/Projects/Endnote/Citavi Attachments/Sulastri, Sulawesty et al. 2015 - Long term monitoring of water.pdf M4 - Citavi

14. Helfrich LA, Smith SA. Fish Kills : Their Causes and Prevention Fish Diseases and Parasites Collecting and. Virginia Coop Ext. 2009; 1–4.

15. Henny C, Nomosatryo S. Changes in water quality and trophic status associated with cage aquaculture in Lake Maninjau , Indonesia. IOP Conf Series: Earth and Environmental Science. 2016. pp. 1–9. doi:10.1088/1755-1315/31/1/012027

16. Wilson J, Abboud S, Beman JM. Primary Production , Community Respiration , and Net Community Production along Oxygen and Nutrient Gradients : Environmental Controls and Biogeochemical Feedbacks within and across “ Marine Lakes .” Front Mar Sci. 2017;4. doi:10.3389/fmars.2017.00012

17. Hoellein TJ, Bruesewitz DA, Richardson DC. Revisiting Odum ( 1956 ): A synthesis of aquatic ecosystem metabolism. Limnol Ocean. 2013;58: 2089–2100. doi:10.4319/lo.2013.58.6.2089

18. Bagarinao TU. Sulfide as a Toxicant in Aquatic Habitats. SEAFDEC Asian Aquac. 1993;XV: 3–5.

19. Sugiarti, Sutamihardja RTM, Citroreksoko P. Distribusi spasial sulfida total di kolom air Danau Maninjau Sumatera Barat. Oseanologi dan Limnol di Indones. 2011;37: 139–154.

20. Henny C, Nomosatryo S. Dinamika Sulfida di Danau maninjau: implikasi terhadap pelepasan fosfat di lapisan hipolimnion. Proceeding of Seminar National Limnologi VI. 2012. pp. 91–106.

21. Luther GW, Glazer BT, Trouwborst R, Shultz BR, Druschel G, Kraiya C. Iron and Sulfur Chemistry in a Strati ed Lake: Evidence for Iron-Rich Sul de Complexes. Aquat Geochemistry. 2003;9: 87–110.

22. North RL, Johansson J, Vandergucht D, Doig LE, Liber K. Evidence for internal phosphorus loading in a large prairie reservoir ( Lake Diefenbaker , Saskatchewan ) Evidence for internal phosphorus loading in a large prairie reservoir ( Lake Diefenbaker , Saskatchewan ). J Great Lakes Res. 2015. doi:10.1016/j.jglr.2015.07.003

23. Nikolai SJ, Dzialowski AR. Limnologica Effects of internal phosphorus loading on nutrient limitation in a eutrophic reservoir. Limnologica. 2014;49: 33–41. doi:10.1016/j.limno.2014.08.005

24. Kisand A, Nõges P. Sediment phosphorus release in phytoplankton dominated versus macrophyte dominated shallow lakes : importance of oxygen conditions. Hydrobiologia. 2003;506–509: 129–133.

25. Niirnberg GK, Mendota L, Sam- L, Lake S, Waubesa L. The prediction of internal phosphorus load in lakes with anoxic hypolimnia ’. Limnol Ocean. 1984;29: 111–124.

26. Solanki VR, Murthy SS, Kaur A, Raja SS. Variations in dissolved oxygen and biochemical oxygen demand in two freshwater lakes of Bodhan, Andhra Pradesh, India. Nat Environ Pollut Technol. 2007;6: 623–628.

27. Aubriot L, Wagner F, Falkner G. The phosphate uptake behaviour of phytoplankton communities in eutrophic lakes reflects alterations in the phosphate supply The phosphate uptake behaviour of phytoplankton communities in eutrophic lakes reflects alterations in the phosphate supply. Eur J Phycol. 2000;35: 255–262. doi:10.1080/09670260010001735851

28. Lv J, Wu H, Chen M. Limnologica Effects of nitrogen and phosphorus on phytoplankton composition and biomass in 15 subtropical , urban shallow lakes in Wuhan , China. Limnologica. 2011;41: 48–56. doi:10.1016/j.limno.2010.03.003

29. Peeters F, Atamanchuk D, Tengberg A, Encinas-ferna J. Lake Metabolism : Comparison of Lake Metabolic Rates Estimated from a Diel CO 2- and the Common Diel O 2- Technique. PLoS One. 2016;11: 1–24. doi:10.1371/journal.pone.0168393

30. Zang C, Huang S, Wu M, Du S, Scholz M, Gao F, et al. Comparison of Relationships Between pH , Dissolved Oxygen and Chlorophyll a for Aquaculture. Water Air Soil Pollut. 2011;219: 157–174. doi:10.1007/s11270-010-0695-3

31. Morgan AM, Royer T V, David MB, Gentry LE. Relationships among Nutrients, Chlorophyll- a , and Dissolved Oxygen in Agricultural Streams in Illinois. J Environ Qual. 2006;35: 1110–1117. doi:10.2134/jeq2005.0433

32. Toan S, Phu P. Research on the Correlation Between Chlorophyll-a and Organic Matter BOD , COD , Phosphorus , and Total Nitrogen in Stagnant Lake Basins. In: Kaneko N, Yoshiura S, Kobayashi M, editors. Sustainable living with environmental risks. 2017. pp. 177–191. doi:10.1007/978-4-431-54804-1

33. Xu Z, Xu YJ. Rapid field estimation of biochemical oxygen demand in a subtropical eutrophic urban lake with chlorophyll a fluorescence. Env Monit Assess. 2015;187: 4170–4184. doi:10.1007/s10661-014-4171-1

34. Carignan R, Hudon C. Influence of diel cycles of respiration , chlorophyll , and photosynthetic parameters on the summer metabolic balance of temperate lakes and rivers. Can J Fish Aquat Sci. 2009;66: 1048–1058. doi:10.1139/F09-058

35. Tonetta D, R. Laudares-Silva, MM. Petrucio. Planktonic production and respiration in a subtropical lake dominated by Cyanobacteria. Braz J Biol. 2015;75: 460–470.

36. Siuda A, Elsi S, Ryszard J. The Relationship between Primary Production and Respiration in the Photic Zone of the Great Mazurian Lakes ( GMLS ), in Relation to Trophic Conditions , Plankton Composition and Other Ecological Factors The relationship between primary production and resp. Pol J Ecol. 2017;65: 303–323. doi:10.3161/15052249PJE2017.65.3.001

37. Amirkolaie AK. Environmental impact of nutrient discharged by aquaculture waste water on the Haraz River. Fish Aquat Sci. 2008;3: 275–279.

38. Marganof. Model Pengendalian Pencemaran. Model pengendalian pencemaran perairan di Danau Maninjau Sumatera Barat. Dissertation. Bogor Agricultural University. 2007.

39. Erlania R, Prasetio AB, Joni H. Dampak manajemen pakan dari kegiatan budiaya ikan nila ( Oreochromis niloticus ) di karamba jaring apung terhadap kualita perairan Danau Maninjau. Pros Forum Inov Teknol Akuakultur. 2010; 621–631.

40. Duan Y, Dong X, Zhang X, Miao Z. Effects of dissolved oxygen concentration and stocking density on the growth , energy budget and body composition of juvenile Japanese flounder , Paralichthys olivaceus ( Temminck et Schlegel ). Aquac Res. 2011;42: 407–416. doi:10.1111/j.1365-2109.2010.02635.x

41. Neto RM, Ostrensky A. Nutrient load estimation in the waste of Nile tilapia Oreochromis niloticus ( L .) reared in cages in tropical climate conditions. Aquac Res. 2015;46: 1309–1322. doi:10.1111/are.12280

# S2 Table. Table of the variable correlation used to conceptualise the model

| Parameters | Model Nodes | Node type | Physical factors | | | | | | | | | | | | | Chemical factors | | | | | | | | Biological factors | | | | | | Antropogenic factors | | | | | | Output | |
| --- | --- | --- | --- | --- | --- | --- | --- | --- | --- | --- | --- | --- | --- | --- | --- | --- | --- | --- | --- | --- | --- | --- | --- | --- | --- | --- | --- | --- | --- | --- | --- | --- | --- | --- | --- | --- | --- |
|  |  |  | S | RI | CD | LI | SSI | WT | WS | WD | WCV | MIX | EZM | AL | AH | DOe | DOm | H2S | Fe | PO4 | BODep | BODmet | Poe | GPPep | GPPmet | Chlo-a ep | Chlo-a met | RRep | RRmet | AFW | OSR | FM | SD | NAC | Feed | MFK | GD |
| Physical factors | Season (S) | Parent |  | ++ | ++ | + | + | + | ++ | 0 | + | + | + | + | 0 | + | + | 0 | 0 | 0 | 0 | 0 | 0 | + | + | + | + | + | + | + | ++ | 0 | ++ | ++ | + | + | + |
|  | Rainfall intensity (RI) | Child | 0 |  | 0 | + | ++ | + | 0 | 0 | 0 | 0 | + | + | 0 | + | + | 0 | 0 | 0 | 0 | 0 | + | + | + | + | + | + | + | + | ++ | 0 | + | + | + | + | + |
|  | Cloudy days (CD) | Child | 0 | + |  | ++ | + | + | 0 | 0 | 0 | 0 | 0 | 0 | 0 | + | + | 0 | 0 | 0 | 0 | 0 | 0 | + | + | + | + | + | + | 0 | 0 | 0 | 0 | 0 | 0 | 0 | 0 |
|  | Light Intensity (LI) | Child | 0 | 0 | 0 |  | ++ | + | 0 | 0 | 0 | + | 0 | 0 | 0 | ++ | ++ | 0 | 0 | 0 | 0 | 0 | 0 | + | + | + | + | 0 | 0 | 0 | 0 | 0 | 0 | 0 | 0 | 0 | 0 |
|  | Schdmidt Stability Index (SSI) | Child | 0 | 0 | 0 | 0 |  | 0 | 0 | 0 | 0 | ++ | 0 | 0 | 0 | 0 | 0 | 0 | 0 | 0 | 0 | 0 | 0 | 0 | 0 | 0 | 0 | 0 | 0 | 0 | 0 | 0 | 0 | 0 | 0 | 0 | 0 |
|  | Water transparency (WT) | Child | 0 | 0 | 0 | ++ | + |  | 0 | 0 | 0 | + | 0 | + | 0 | + | + | 0 | 0 | 0 | 0 | 0 | 0 | + | + | + | + | 0 | 0 | 0 | 0 | 0 | 0 | 0 | 0 | + | 0 |
|  | Wind speed (WS) | Child | 0 | + | + | 0 | 0 | 0 |  | 0 | ++ | ++ | 0 | 0 | 0 | 0 | 0 | 0 | 0 | 0 | 0 | 0 | 0 | 0 | 0 | 0 | 0 | 0 | 0 | + | 0 | 0 | 0 | 0 | 0 | + | + |
|  | Wind Direction (WD) | Parent | 0 | 0 | 0 | 0 | 0 | 0 | 0 |  | 0 | 0 | 0 | + | 0 | 0 | 0 | 0 | 0 | 0 | 0 | 0 | 0 | 0 | 0 | 0 | 0 | 0 | 0 | ++ | + | 0 | 0 | 0 | 0 | ++ | 0 |
|  | Water current velocity (WCV) | Child | 0 | 0 | 0 | 0 | 0 | 0 | 0 | 0 |  | + | 0 | 0 | 0 | 0 | 0 | 0 | 0 | 0 | 0 | 0 | 0 | 0 | 0 | 0 | 0 | 0 | 0 | ++ | + | 0 | 0 | 0 | 0 | 0 | 0 |
|  | Mixing (MIX) | Child | 0 | 0 | 0 | 0 | 0 | 0 | 0 | 0 | 0 |  | 0 | + | 0 | 0 | + | + | + | + | + | + | + | + | + | + | + | 0 |  | 0 | 0 | 0 | + | + | 0 | ++ | ++ |
|  | Epilimnion zone after mixing (EZM) | Child | 0 | 0 | 0 | 0 | 0 | 0 | 0 | 0 | 0 | 0 |  | 0 | 0 | 0 | 0 | 0 | 0 | 0 | 0 | 0 | 0 | 0 | 0 | 0 | 0 | 0 | 0 | 0 | 0 | 0 | 0 | 0 | 0 | ++ | ++ |
|  | Anoxic layer (AL) | Child | 0 | 0 | 0 | 0 | 0 | 0 | 0 | 0 | 0 | 0 | ++ |  | 0 | + | + | 0 | 0 | + | 0 | 0 | 0 | 0 | 0 | 0 | 0 | 0 | 0 | 0 | 0 | 0 | 0 | 0 | 0 | + | + |
|  | Anoxic Hypolimnion (AH) | Parent | 0 | 0 | 0 | 0 | 0 | 0 | 0 | 0 | 0 | 0 | 0 | 0 |  | 0 | 0 | + | ++ | ++ | 0 | 0 | 0 | 0 | 0 | 0 | 0 | 0 | 0 | 0 | 0 | 0 | 0 | 0 | 0 | 0 | 0 |
| Chemical factors | DO epilimnion (DOe) | Child | 0 | 0 | 0 | 0 | 0 | 0 | 0 | 0 | 0 | 0 | + | + | 0 |  | + | 0 | 0 | 0 | + | + | 0 | ++ | 0 | 0 | 0 | 0 | 0 | 0 | 0 | ++ | ++ | ++ | 0 | 0 | 0 |
|  | DO metalimnetic (DOm) | Child | 0 | 0 | 0 | 0 | 0 | 0 | 0 | 0 | 0 | 0 | 0 | + | 0 | 0 |  | + | 0 | ++ | 0 | 0 | 0 | 0 | 0 | 0 | 0 | ++ | ++ | 0 | 0 | 0 | 0 | 0 | 0 | ++ | + |
|  | H_2_S | Child | 0 | 0 | 0 | 0 | 0 | 0 | 0 | 0 | 0 | 0 | 0 | 0 | 0 | 0 | 0 |  | 0 | ++ | 0 | 0 | 0 | 0 | 0 | 0 | 0 | 0 | 0 | 0 | 0 | 0 | 0 | 0 | 0 | ++ | ++ |
|  | Fe concentration (Fe) | Parent | 0 | 0 | 0 | 0 | 0 | 0 | 0 | 0 | 0 | 0 | 0 | 0 | 0 | 0 | 0 | -- |  | -- | 0 | 0 | + | 0 | 0 | 0 | 0 | 0 | 0 | 0 | 0 | 0 | 0 | 0 | 0 | 0 | 0 |
|  | PO4 Released from sediment (PO4) | Child | 0 | 0 | 0 | 0 | 0 | 0 | 0 | 0 | 0 | 0 | 0 | 0 | 0 | 0 | 0 | 0 | 0 |  | 0 | 0 | ++ | + | + | + | ++ | + | + | 0 | 0 | 0 | 0 | 0 | 0 | + | + |
|  | BOD epilimnion (BODep) | Child | 0 | 0 | 0 | 0 | 0 | 0 | 0 | 0 | 0 | 0 | + | + | 0 | -- | - | 0 | 0 | 0 |  | 0 | 0 | + | + | 0 | 0 | 0 | 0 | 0 | 0 | 0 | 0 | 0 | 0 | 0 | 0 |
|  | BOD metalimnion (BODmet) | Child | 0 | 0 | 0 | 0 | 0 | 0 | 0 | 0 | 0 | 0 | + | + | 0 | - | -- | 0 | 0 | 0 | 0 |  | 0 | 0 | + | 0 | + | 0 | 0 | 0 | 0 | 0 | 0 | 0 | 0 | + | + |
|  | PO4 concentration epilimnion (POe) | Child | 0 | 0 | 0 | 0 | 0 | 0 | 0 | 0 | 0 | 0 | 0 | + | 0 | + | + | 0 | 0 | 0 | 0 | 0 |  | + | + | ++ | + | 0 | 0 | 0 | 0 | 0 | 0 | 0 | 0 | 0 | 0 |
| Biological factors | GPP epilimnion (GPPep) | Child | 0 | 0 | 0 | 0 | 0 | 0 | 0 | 0 | 0 | 0 | + | ++ | 0 | + | + | 0 | 0 | + | + | + | 0 |  | 0 | 0 | 0 | 0 | 0 | 0 | 0 | 0 | 0 | 0 | 0 | 0 | 0 |
|  | GPP metalimnion(GPPmet) | Child | 0 | 0 | 0 | 0 | 0 | 0 | 0 | 0 | 0 | 0 | + | ++ | 0 | 0 | + | 0 | 0 | + | + | + | 0 | 0 |  | 0 | 0 | 0 | 0 | 0 | 0 | 0 | 0 | 0 | 0 | 0 | 0 |
|  | Chlo-a epilimnion (Chlo-a ep) | Child | 0 | 0 | 0 | 0 | 0 | ++ | 0 | 0 | 0 | 0 | + | + | 0 | ++ | + | 0 | 0 | 0 | ++ | + | + | ++ | + |  | 0 | + | + | 0 | 0 | + | 0 | 0 | + | + | + |
|  | Chlo-a metalimnion (Chlo-a met) | Child | 0 | 0 | 0 | 0 | 0 | 0 | 0 | 0 | 0 | 0 | - | - | 0 | 0 | ++ | 0 | 0 | 0 | ++ | ++ | 0 | + | + | 0 |  | + | + | 0 | 0 | + | 0 | 0 | 0 | + | + |
|  | Respiration rate epilimnion (RRep) | Child | 0 | 0 | 0 | 0 | 0 | 0 | 0 | 0 | 0 | 0 | 0 | 0 | 0 | - | - | 0 | 0 | 0 | 0 | 0 | 0 | -- | - | 0 | 0 |  | + | 0 | 0 | 0 | 0 | 0 | 0 | + | + |
|  | Respiration rate metalinion (RRmet) | Child | 0 | 0 | 0 | 0 | 0 | 0 | 0 | 0 | 0 | 0 | 0 | 0 | 0 | - | -- | 0 | 0 | 0 | 0 | 0 | 0 | + | ++ | 0 | 0 | 0 |  | 0 | 0 | 0 | 0 | 0 | 0 | + | + |
| Antropogenic factors | Accumulated fish waste (AFW) | Child | 0 | 0 | 0 | 0 | 0 | 0 | 0 | 0 | 0 | + | + | + | + | -- | -- | - | - | -- | ++ | ++ | + | - | - | + | + | 0 | 0 |  | 0 | -- | -- | 0 | 0 | + | + |
|  | Organic sediment run off (OSR) | Child | 0 | 0 | 0 | 0 | 0 | 0 | 0 | 0 | 0 | 0 | 0 | + | 0 | -- | -- | 0 | 0 | 0 | ++ | ++ | ++ | - | - | + | + | 0 | 0 | 0 |  | 0 | 0 | 0 | 0 | + | + |
|  | Feeding management (FM) | Child | 0 | 0 | 0 | 0 | 0 | 0 | 0 | 0 | 0 | 0 | + | + | 0 | + | + | 0 | 0 | + | + | + | + | 0 | 0 | + | + | 0 | 0 | ++ | 0 |  | 0 | 0 | ++ | + | + |
|  | Stocking density (SD) | Child | 0 | 0 | 0 | 0 | 0 | 0 | 0 | 0 | 0 | 0 | 0 | 0 | 0 | + | + | 0 | 0 | + | + | + | + | + | + | + | + | ++ | + | + | 0 | + |  | + | ++ | + | 0 |
|  | Number of active cages (NAC) | Child | 0 | 0 | 0 | 0 | 0 | 0 | 0 | 0 | 0 | 0 | 0 | + | 0 | + | + | 0 | 0 | + | ++ | ++ | + | ++ | ++ | + | + | ++ | ++ | ++ | 0 | ++ | ++ |  | ++ | + | + |
|  | Feed | Child | 0 | 0 | 0 | 0 | 0 | + | 0 | 0 | 0 | 0 | + | + | 0 | 0 | 0 | 0 | 0 | + | + | + | + | 0 | 0 | + | + | 0 | 0 | ++ | 0 | 0 | 0 | 0 |  | + | + |
| Output | IWCCF Fish Mass fish kills (MFK) | Child | 0 | 0 | 0 | 0 | 0 | 0 | 0 | 0 | 0 | 0 | 0 | + | - | + | + | 0 | 0 | + | + | + | + | + | + | + | + | 0 | 0 | 0 | 0 | 0 | 0 | 0 | + |  | + |
|  | *Gobiopterus* disappearance (GD) | Outcome | 0 | 0 | 0 | 0 | 0 | 0 | 0 | 0 | 0 | 0 | 0 | 0 | - | 0 | 0 | 0 | 0 | 0 | 0 | 0 | 0 | 0 | 0 | 0 | 0 | 0 | 0 | 0 | 0 | 0 | 0 | 0 | 0 | 0 |  |

# S3 Table. Methods to fill CPTs, CPTs before and after being compiled

| Node | Type of Node | Method to fill CPT Tables | Data Source (s) | Type of data in CPT (Chance (%)/ deterministic) | CPT before being compiled | CPT after being compiled |
| --- | --- | --- | --- | --- | --- | --- |
| Season | Parent | Data | BMKG data (2000-2010) | Chance | 16.7  83.3 | 16.7  83.3 |
| Rainfall  intensity | Child | Data | BMKG data (2000-2010) (n=3,650) | Chance | Dry season:  No rain: 76  Light rain 3  Moderate rain: 6  Heavy rain: 15 | No rain: 52.7  Light rain 10.5  Moderate rain:9.33  Heavy rain:27.5 |
|  |  |  |  |  | Wet season  No rain:48  Light rain:12  Moderate rain:10  Heavy rain:30 |  |
| Cloudy | Child | Data | BMKG data (2000-2010) | Chance | Dry Season:  Yes:20  No:80 | Yes: 70  No:30 |
|  |  |  |  |  | Wet season:  Yes:80  No:20 |  |
| Light intensity | Child | Expert knowledge | Based on solar radiation data from BMKG (2000-2010) | Chance | Cloudy (Yes)-Water transparency (Low):  High: 30, Medium:35; Low:35 | High: 51.2  Medium:38  Low:10.8 |
|  |  |  |  |  | Cloudy (Yes)-Water transparency (High): High:45, Medium:50, Low:5 |  |
|  |  |  |  |  | Cloudy (No)-Water transparency (Low): High:45, Medium:45, Low:10 |  |
|  |  |  |  |  | Cloudy (No)-Water transparency (High): High:90, Medium:10, Low:0 |  |
| Windspeed | Child | Data | BMKG data (2000-2010) (n=3,650) | Chance | Dry season:  Calm:42.3  Moderate:19  Strong:25.4  Gale-storm:13.3 | Calm: 52.6  Moderate: 18.2  Strong:20  Gale-storm:9.22 |
|  |  |  |  |  | Wet season:  Calm: 54.7  Moderate: 18  Strong: 18.9  Gale-storm:8.4 |  |
| Mixing | Child | Expert knowledge | Based on criteria of environmental condition to trigger upwelling from Ministry of Marine and Fisheries verified with data from Fisheries Agency and RCL-IIOS | Chance | SSI (High)-Windspeed (calm): Yes:0, No:100 | Yes: 24.1  No:75.9 |
|  |  |  |  |  | SSI (High)-Windspeed (moderate): Yes:15, No:85 |  |
|  |  |  |  |  | SSI (High)-Windspeed (strong): Yes:40, No:60 |  |
|  |  |  |  |  | SSI (High)-Windspeed (Gale-storm): Yes:50, No:50 |  |
|  |  |  |  |  | SSI (Medium)-Windspeed (calm): Yes:15, No:85 |  |
|  |  |  |  |  | SSI (Medium)-Windspeed (Moderate): Yes:30, No:70 |  |
|  |  |  |  |  | SSI (Medium)-Windspeed (Strong): Yes:30, No:70 |  |
|  |  |  |  |  | SSI (Medium)-Windspeed (Gale-storm): Yes:50, No:50 |  |
|  |  |  |  |  | SSI (Low)-Windspeed (calm): Yes:20, No:80 |  |
|  |  |  |  |  | SSI (Low)-Windspeed (moderate): Yes:45, No:55 |  |
|  |  |  |  |  | SSI (Low)-Windspeed (moderate): Yes:45, No:55 |  |
|  |  |  |  |  | SSI (Low)-Windspeed (strong): Yes:65, No:35 |  |
|  |  |  |  |  | SSI (Low)-Windspeed (Gale-storm): Yes:100, No:0 |  |
| Wind Direction | Parent | Data | RCL-IIOS data (2018) (n=10,926) | Chance | North:4.8, NorthEast:21.8, East:36.3, SouthEast:0, Other direction:37.1 | North:4.8  NorthEast:21.8  East:36.3, SouthEast:0  Other direction:37.1 |
| Water transparency | Child | Data | RCL-IIOS and Setiawan *et al*.(2019) | Chance | Chlorophyll-a (High): Low:24, High:76 | Low: 30.3  High:69.7 |
|  |  |  |  |  | Chlorophyll-a (Medium): Low:52, High:48 |  |
|  |  |  |  |  | Chlorophyll-a (Medium): Low:15, High:85 |  |
| Anoxic layer | Child | Expert knowledge | Based on data RCL-IIOS’s dissolved oxygen data (2001-2014) | Chance | GPP epilimnion (High)-GPP Metalimnion (High): wide: 0, narrow: 100 | wide: 74  narrow: 26 |
|  |  |  |  |  | GPP epilimnion (High)-GPP Metalimnion (Low): wide: 60, narrow: 40 |  |
|  |  |  |  |  | GPP epilimnion (High)-GPP Metalimnion (Low): wide: 50, narrow: 50 |  |
|  |  |  |  |  | GPP epilimnion (High)-GPP Metalimnion (Low): wide: 1000, narrow: 0 |  |
| Epilimnion zone after mixing | Child | Expert knowledge |  | Chance | Anoxic layer (wide)-Mixing(yes): Anoxic:100, Oxic:0 | Anoxic:21  Oxic:79 |
|  |  |  |  |  | Anoxic layer (wide)-Mixing(no): Anoxic:0, Oxic:100 |  |
|  |  |  |  |  | Anoxic layer (Narrow)-Mixing (Yes): Anoxic:50, Oxic:50 |  |
|  |  |  |  |  | Anoxic layer (Narrow)-Mixing (no): Anoxic:0, Oxic:100 |  |
| SSI | Child | Data | Calculated from high frequency measurement data by RCL-IIOS (2014-2017) (n=540) | Chance | Light intensity (High)-Rainfall intensity (No rain): High:100, Medium:0, Low:0 | High:50.3, Medium:23.2  Low:26.5 |
|  |  |  |  |  | Light intensity (High)-Rainfall intensity (moderate rain): High:0, Medium:50, Low:50 |  |
|  |  |  |  |  | Light intensity (High)-Rainfall intensity (Heavy rain): High:0, Medium:30, Low:70 |  |
|  |  |  |  |  | Light intensity (Medium)-Rainfall intensity (No rain): High:60, Medium:40, Low:0 |  |
|  |  |  |  |  | Light intensity (Medium)-Rainfall intensity (Light rain): High:70, Medium:30, Low:0 |  |
|  |  |  |  |  | Light intensity (Medium)-Rainfall intensity (Moderate rain): High:0, Medium:40, Low:60 |  |
|  |  |  |  |  | Light intensity (Medium)-Rainfall intensity (Heavy rain): High:0, Medium:20, Low:80 |  |
|  |  |  |  |  | Light intensity (Low)-Rainfall intensity (No rain): High:50, Medium:50, Low: 0 |  |
|  |  |  |  |  | Light intensity (Low)-Rainfall intensity (light rain): High:50, Medium:50, Low: 0 |  |
|  |  |  |  |  | Light intensity (Low)-Rainfall intensity (moderate rain): High:0, Medium:30, Low: 70 |  |
|  |  |  |  |  | Light intensity (Low)-Rainfall intensity (Heavy rain): High:0, Medium:0, Low: 100 |  |
| Water current velocity | Child | Data | RCL-IIOS combined with data from BMKG | Chance | Windspeed (Calm): fast:5, Medium:15, Slow:80 | Fast:30.6  Medium:14.6  Slow:54.8 |
|  |  |  |  |  | Windspeed (Calm): fast:15, Medium:15, Slow:70 |  |
|  |  |  |  |  | Windspeed (Calm): fast:80, Medium:20, Slow:10 |  |
|  |  |  |  |  | Windspeed (Calm): fast:100, Medium:0, Slow:0 |  |
| Anoxic hypolimnion | Child | Expert knowledge | Based on Henny and Sulung Nomosatryo (2012) Sulastri (2002) | Chance | Yes:90  No:10 | Yes:90  No:10 |
| BOD epilimnion | Child | Expert knowledge | Based on patchy data from Ministry of Public Works and Erlania,Prasetio, & Joni (2010) | Chance | Organic sediment run-off (High)-Accumulated fish waste (High)-Chlo-a metalimnion (High): High:100, Low:0 | High:73.7  Low:26.3 |
|  |  |  |  |  | Organic sediment run-off (High)-Accumulated fish waste (Low)-Chlo-a metalimnion (High): High:75, Low:25 |  |
|  |  |  |  |  | Organic sediment run-off (High)-Accumulated fish waste (Low)-Chlo-a metalimnion (Low): High:60, Low:40 |  |
|  |  |  |  |  | Organic sediment run-off (Medium)-Accumulated fish waste (Low)-Chlo-a metalimnion (Low): High:90, Low:10 |  |
|  |  |  |  |  | Organic sediment run-off (Medium)-Accumulated fish waste (High)-Chlo-a metalimnion (Low): High:85, Low:15 |  |
|  |  |  |  |  | Organic sediment run-off (Medium)-Accumulated fish waste (Low)-Chlo-a metalimnion (High): High:75, Low:25 |  |
|  |  |  |  |  | Organic sediment run-off (Medium)-Accumulated fish waste (Low)-Chlo-a metalimnion (Low): High:60, Low:40 |  |
|  |  |  |  |  | Organic sediment run-off (Low)-Accumulated fish waste (High)-Chlo-a metalimnion (High): High:85, Low:15 |  |
|  |  |  |  |  | Organic sediment run-off (Low)-Accumulated fish waste (High)-Chlo-a metalimnion (Low): High:60, Low:40 |  |
|  |  |  |  |  | Organic sediment run-off (Low)-Accumulated fish waste (Low)-Chlo-a metalimnion (High): High:65, Low:35 |  |
|  |  |  |  |  | Organic sediment run-off (Low)-Accumulated fish waste (Low)-Chlo-a metalimnion (Low): High:20, Low:80 |  |
| PO4 Concentration Epilimnion | Child | Expert knowledge | Based on patchy data of Total Phosphate data from RCL-IIOS (2001-2014) | Chance | Organic sediment run-off (High)-PO4 released from sediment (High): High:60, Low: 40 | High:52  Low:48 |
|  |  |  |  |  | Organic sediment run-off (High)-PO4 released from sediment Low): High:40, Low:60 |  |
|  |  |  |  |  | Organic sediment run-off (Medium)-PO4 released from sediment (High): High:40, Low:60 |  |
|  |  |  |  |  | Organic sediment run-off (Medium)-PO4 released from sediment (Low): High:40, Low:60 |  |
|  |  |  |  |  | Organic sediment run-off (Low)-PO4 released from sediment (High): High:60, Low:40 |  |
|  |  |  |  |  | Organic sediment run-off (Low)-PO4 released from sediment (Low): High:10, Low:90 |  |
| Chlo-a epilimnion | Child | Data | RCL-IIOS (2001-2014), Henny and Sulung Nomosatryo (2012) | Chance | PO4 Concentration epilimnion (High)-PO4 released from sediment (High): High:80, Medium:15, Low:5 | High:75.8  Medium:12.9  Low:11.4 |
|  |  |  |  |  | PO4 Concentration epilimnion (High)-PO4 released from sediment (Low): High:80, Medium:15, Low:5 |  |
|  |  |  |  |  | PO4 Concentration epilimnion (Low)-PO4 released from sediment (High): High:80, Medium:10, Low:10 |  |
|  |  |  |  |  | PO4 Concentration epilimnion (Low)-PO4 released from sediment (Low): High:5, Medium:15, Low:80 |  |
| Respiration rate epilimnion | Child | Expert knowledge | Based on one-time data measurement from RCL-IIOS |  | Stocking density (High): High:90, Low:10 | High:63.7  Low:36.3 |
|  |  |  |  |  | Stocking density (Medium): High:70, Low:30 |  |
|  |  |  |  |  | Stocking density (Low): High:50, Low:50 |  |
| Respiration rate metalimnion | Child | Expert knowledge | Based on one-time data measurement from RCL-IIOS | Chance | Chlo-a metalimnion (High): High: 80, Low:20 | High: 50  Low:50 |
|  |  |  |  |  | Chlo-a metalimnion (Low): High: 20, Low:80 |  |
| BOD metalimnion | Child | Expert knowledge | Based on the paper of Junaidi, Syandri, and Azrita (2014); Erlania,Prasetio, & Joni (2010); Ministry of Public Work (2013) | Chance | Chlo-a metalimnion (High)-Organic sediment runoff (High)- Accumulated fish waste (High): High:100, Low:0 | High:75.2  Low:24.6 |
|  |  |  |  |  | Chlo-a metalimnion (High)-Organic sediment runoff (High)- Accumulated fish waste (Low): High:75, Low:25 |  |
|  |  |  |  |  | Chlo-a metalimnion (High)-Organic sediment runoff (Medium)- Accumulated fish waste (High): High:90, Low:10 |  |
|  |  |  |  |  | Chlo-a metalimnion (High)-Organic sediment runoff (Medium)- Accumulated fish waste (Low): High:75, Low:25 |  |
|  |  |  |  |  | Chlo-a metalimnion (High)-Organic sediment runoff (Low)- Accumulated fish waste (Low): High:80, Low:20 |  |
|  |  |  |  |  | Chlo-a metalimnion (Low)-Organic sediment runoff (High)- Accumulated fish waste (High): High:75, Low:25 |  |
|  |  |  |  |  | Chlo-a metalimnion (Low)-Organic sediment runoff (High)- Accumulated fish waste (Low): High:60, Low:40 |  |
|  |  |  |  |  | Chlo-a metalimnion (Low)-Organic sediment runoff (Medium)- Accumulated fish waste (High): High:85, Low:15 |  |
|  |  |  |  |  | Chlo-a metalimnion (Low)-Organic sediment runoff (Medium)- Accumulated fish waste (Low): High:60, Low:40 |  |
|  |  |  |  |  | Chlo-a metalimnion (Low)-Organic sediment runoff (Low)- Accumulated fish waste (High): High:80, Low:20 |  |
|  |  |  |  |  | Chlo-a metalimnion (Low)-Organic sediment runoff (Low)- Accumulated fish waste (Low): High:20, Low:80 |  |
| PO4 Released from hypolimnion | Child | Expert knowledge | Based on the paper of Henny and Sulung Nomosatryo (2012) and Nikolai & Dzialowski (2014) | Chance | Fe^2+^ concentration (Low)-Anoxic hypolimnion (yes): High:100, Low:0 | High:92  Low:8 |
|  |  |  |  |  | Fe^2+^ concentration (Low)-Anoxic hypolimnion (no): High:20, Low:80 |  |
| Reactive Fe (Fe^2+^) Concentration | Parent | Expert knowledge | Based on the paper of Henny and Sulung Nomosatryo (2012) | Deterministic | Low | Low |
| Chlo-a metalimnion | Child | Expert knowledge | Based on Henny and Sulung Nomosatryo (2012) | Chance | PO_4_ released from hypolimnion (High): High:80. Low: 20 | High: 75.2  Low: 25.8 |
|  |  |  |  |  | PO_4_ released from hypolimnion (Low): High:20. Low: 80 |  |
| H_2_S | Child | Data | Sugiarti, Sutamihardja, and Citroreksoko (2011) | Chance | Fe^2+^ concentration (Low)-Anoxic hypolimnion (yes): High:0, Low:0 | High:93  Low:7 |
|  |  |  |  |  | Fe^2+^ concentration (Low)-Anoxic hypolimnion (no): High:30, Low70 |  |
| GPP epilimnion | Child | Expert knowledge | Based on one-time data measurement from RCL-IIOS | Chance | Respiration rate epilimnion (High)-DO epilimnion (High): High:50, Low:50 | High:42.8  Low:57.2 |
|  |  |  |  |  | Respiration rate epilimnion (High)-DO epilimnion (Medium): High:30, Low:70 |  |
|  |  |  |  |  | Respiration rate epilimnion (High)-DO epilimnion (Low): High:10, Low:90 |  |
|  |  |  |  |  | Respiration rate epilimnion (Low)-DO epilimnion (High): High:90, Low:10 |  |
|  |  |  |  |  | Respiration rate epilimnion (Low)-DO epilimnion (Medium): High:70, Low:30 |  |
|  |  |  |  |  | Respiration rate epilimnion (Low)-DO epilimnion (Low): High:20, Low:80 |  |
| GPP metalimnion | Child | Expert knowledge | Based on one-time data measurement from RCL-IIOS | Chance | Respiration rate metalimnion (High)-DO metalimnion (High): High:70, Low:30 | High: 23.9  Low:76.1 |
|  |  |  |  |  | Respiration rate metalimnion (High)-DO metalimnion (Very low): High: 0, Low:100 |  |
|  |  |  |  |  | Respiration rate metalimnion (High)-DO metalimnion (Medium): High:50, Low:50 |  |
|  |  |  |  |  | Respiration rate metalimnion (High)-DO metalimnion (Low): High:50, Low:50 |  |
|  |  |  |  |  | Respiration rate metalimnion (High)-DO metalimnion (Low): High:10, Low:90 |  |
|  |  |  |  |  | Respiration rate metalimnion (Low)-DO metalimnion (High): High:80, Low:20 |  |
|  |  |  |  |  | Respiration rate metalimnion (Low)-DO metalimnion (Very Low): High:0, Low:100 |  |
|  |  |  |  |  | Respiration rate metalimnion (Low)-DO metalimnion (Medium): High:30, Low:70 |  |
|  |  |  |  |  | Respiration rate metalimnion (Low)-DO metalimnion (Low): High:0, Low:100 |  |
| DO epilimnion | Child | Expert knowledge | Based on data from RCL-IIOS (2005-2018) | Chance | Light intensity (High)-Chlo-a epilimnion (High)-BOD epilimnion (High): High: 40, Medium:30, Low: 30 | High: 41.4  Medium:26.3  Low: 32.3 |
|  |  |  |  |  | Light intensity (High)-Chlo-a epilimnion (High)-BOD epilimnion (Low): High: 90, Medium: 5, Low: 5 |  |
|  |  |  |  |  | Light intensity (High)-Chlo-a epilimnion (Medium)-BOD epilimnion (High): High: 40, Medium: 30, Low: 30 |  |
|  |  |  |  |  | Light intensity (High)-Chlo-a epilimnion (Medium)-BOD epilimnion (Low): High: 60, Medium: 30, Low: 10 |  |
|  |  |  |  |  | Light intensity (High)-Chlo-a epilimnion (Low)-BOD epilimnion (High): High: 10, Medium: 20, Low: 70 |  |
|  |  |  |  |  | Light intensity (High)-Chlo-a epilimnion (Low)-BOD epilimnion (Low): High: 80, Medium: 10, Low: 10 |  |
|  |  |  |  |  | Light intensity (Medium)-Chlo-a epilimnion (High)-BOD epilimnion (High): High: 30, Medium: 30, Low: 40 |  |
|  |  |  |  |  | Light intensity (Medium)-Chlo-a epilimnion (High)-BOD epilimnion (Low): High: 70, Medium: 20, Low: 10 |  |
|  |  |  |  |  | Light intensity (Medium)-Chlo-a epilimnion (Medium)-BOD epilimnion (High): High: 40, Medium: 40, Low: 20 |  |
|  |  |  |  |  | Light intensity (Medium)-Chlo-a epilimnion (Medium)-BOD epilimnion (Low): High: 50, Medium: 30, Low: 20 |  |
|  |  |  |  |  | Light intensity (Medium)-Chlo-a epilimnion (Low)-BOD epilimnion (High): High: 10, Medium: 10, Low: 80 |  |
|  |  |  |  |  | Light intensity (Medium)-Chlo-a epilimnion (Low)-BOD epilimnion (Low): High: 70, Medium: 20, Low: 10 |  |
|  |  |  |  |  | Light intensity (Low)-Chlo-a epilimnion (High)-BOD epilimnion (High): High: 15, Medium: 25, Low: 60 |  |
|  |  |  |  |  | Light intensity (Low)-Chlo-a epilimnion (High)-BOD epilimnion (Low): High: 20, Medium: 40, Low: 40 |  |
|  |  |  |  |  | Light intensity (Low)-Chlo-a epilimnion (Medium)-BOD epilimnion (High): High: 10, Medium: 20, Low: 70 |  |
|  |  |  |  |  | Light intensity (Low)-Chlo-a epilimnion (Medium)-BOD epilimnion (Low): High: 10, Medium: 20, Low: 70 |  |
|  |  |  |  |  | Light intensity (Low)-Chlo-a epilimnion (Low)-BOD epilimnion (High): High: 80, Medium: 10, Low: 10 |  |
|  |  |  |  |  | Light intensity (Low)-Chlo-a epilimnion (Medium)-BOD epilimnion (Low): High: 10, Medium: 20, Low: 70 |  |
|  |  |  |  |  | Light intensity (Low)-Chlo-a epilimnion (Low)-BOD epilimnion (Low): High: 10, Medium: 10, Low: 80 |  |
| DO metalimnion | Child | Expert knowledge | Based on data from RCL-IIOS (2005-2018) | Chance | Chlorophyll-a metalimnion (High)-BOD Metalimnion (High)-Light intensity (High)-DO epilimnion (High): High :10, Very Low :5, Medium :20, Low :65 | High:16.7  Medium:19.0  Low:59.0  Very Low:5.26 |
|  |  |  |  |  | Chlorophyll-a metalimnion (High)-BOD Metalimnion (High)-Light intensity (High)-DO epilimnion (Medium): High :10, Very Low :5, Medium :20, Low :65 |  |
|  |  |  |  |  | Chlorophyll-a metalimnion (High)-BOD Metalimnion (High)-Light intensity (High)-DO epilimnion (Low): High :10, Very Low :5, Medium :20, Low :65 |  |
|  |  |  |  |  | Chlorophyll-a metalimnion (High)-BOD Metalimnion (High)-Light intensity (Medium)-DO epilimnion (High): High :10, Very Low :5, Medium :20, Low :65 |  |
|  |  |  |  |  | Chlorophyll-a metalimnion (High)-BOD Metalimnion (High)-Light intensity (Medium)-DO epilimnion (Medium): High :10, Very Low :5, Medium :20, Low :65 |  |
|  |  |  |  |  | Chlorophyll-a metalimnion (High)-BOD Metalimnion (High)-Light intensity (Medium)-DO epilimnion (Low): High :10, Very Low :5, Medium :20, Low :65 |  |
|  |  |  |  |  | Chlorophyll-a metalimnion (High)-BOD Metalimnion (High)-Light intensity (Low)-DO epilimnion (High): High :10, Very Low :5, Medium :20, Low :65 |  |
|  |  |  |  |  | Chlorophyll-a metalimnion (High)-BOD Metalimnion (High)-Light intensity (Low)-DO epilimnion (High): High :10, Very Low :5, Medium :20, Low :65 |  |
|  |  |  |  |  | Chlorophyll-a metalimnion (High)-BOD Metalimnion (High)-Light intensity (Low)-DO epilimnion (Low): High :10, Very Low :5, Medium :20, Low :65 |  |
|  |  |  |  |  | Chlorophyll-a metalimnion (High)-BOD Metalimnion (Low)-Light intensity (High)-DO epilimnion (High): High :90, Very Low :0, Medium :10, Low :0 |  |
|  |  |  |  |  | Chlorophyll-a metalimnion (High)-BOD Metalimnion (Low)-Light intensity (High)-DO epilimnion (Medium): High :90, Very Low :0, Medium :10, Low :0 |  |
|  |  |  |  |  | Chlorophyll-a metalimnion (High)-BOD Metalimnion (Low)-Light intensity (High)-DO epilimnion (Low): High :90, Very Low :0, Medium :10, Low :0 |  |
|  |  |  |  |  | Chlorophyll-a metalimnion (High)-BOD Metalimnion (Low)-Light intensity (Medium)-DO epilimnion (High): High :90, Very Low :0, Medium :10, Low :0 |  |
|  |  |  |  |  | Chlorophyll-a metalimnion (High)-BOD Metalimnion (Low)-Light intensity (Medium)-DO epilimnion (Medium): High :90, Very Low :0, Medium :10, Low :0 |  |
|  |  |  |  |  | Chlorophyll-a metalimnion (High)-BOD Metalimnion (Low)-Light intensity (Medium)-DO epilimnion (Low): High :90, Very Low :0, Medium :10, Low :0 |  |
|  |  |  |  |  | Chlorophyll-a metalimnion (High)-BOD Metalimnion (Low)-Light intensity (Low)-DO epilimnion (High): High :90, Very Low :0, Medium :10, Low :0 |  |
|  |  |  |  |  | Chlorophyll-a metalimnion (High)-BOD Metalimnion (Low)-Light intensity (Low)-DO epilimnion (Medium): High :90, Very Low :0, Medium :10, Low :0 |  |
|  |  |  |  |  | Chlorophyll-a metalimnion (High)-BOD Metalimnion (Low)-Light intensity (Low)-DO epilimnion (Low): High :90, Very Low :0, Medium :10, Low :0 |  |
|  |  |  |  |  | Chlorophyll-a metalimnion (Low)-BOD Metalimnion (High)-Light intensity (High)-DO epilimnion (High): High :0, Very Low :10, Medium :10, Low :80 |  |
|  |  |  |  |  | Chlorophyll-a metalimnion (Low)-BOD Metalimnion (High)-Light intensity (High)-DO epilimnion (Medium): High :0, Very Low :10, Medium :10, Low :80 |  |
|  |  |  |  |  | Chlorophyll-a metalimnion (Low)-BOD Metalimnion (High)-Light intensity (High)-DO epilimnion (Low): High :0, Very Low :10, Medium :10, Low :80 |  |
|  |  |  |  |  | Chlorophyll-a metalimnion (Low)-BOD Metalimnion (High)-Light intensity (Medium)-DO epilimnion (High): High :0, Very Low :10, Medium :10, Low :80 |  |
|  |  |  |  |  | Chlorophyll-a metalimnion (Low)-BOD Metalimnion (High)-Light intensity (Medium)-DO epilimnion (Low): High :0, Very Low :10, Medium :10, Low :80 |  |
|  |  |  |  |  | Chlorophyll-a metalimnion (Low)-BOD Metalimnion (High)-Light intensity (Low)-DO epilimnion (High): High :0, Very Low :10, Medium :10, Low :80 |  |
|  |  |  |  |  | Chlorophyll-a metalimnion (Low)-BOD Metalimnion (High)-Light intensity (Low)-DO epilimnion (Medium): High :0, Very Low :10, Medium :10, Low :80 |  |
|  |  |  |  |  | Chlorophyll-a metalimnion (Low)-BOD Metalimnion (High)-Light intensity (Low)-DO epilimnion (Low): High :0, Very Low :10, Medium :10, Low :80 |  |
|  |  |  |  |  | Chlorophyll-a metalimnion (Low)-BOD Metalimnion (Low)-Light intensity (High)-DO epilimnion (High): High :5, Very Low :5, Medium :40, Low :50 |  |
|  |  |  |  |  | Chlorophyll-a metalimnion (Low)-BOD Metalimnion (Low)-Light intensity (High)-DO epilimnion (Medium): High :5, Very Low :5, Medium :40, Low :50 |  |
|  |  |  |  |  | Chlorophyll-a metalimnion (Low)-BOD Metalimnion (Low)-Light intensity (High)-DO epilimnion (Low): High :5, Very Low :5, Medium :40, Low :50 |  |
|  |  |  |  |  | Chlorophyll-a metalimnion (Low)-BOD Metalimnion (Low)-Light intensity (Medium)-DO epilimnion (High): High :5, Very Low :5, Medium :40, Low :50 |  |
|  |  |  |  |  | Chlorophyll-a metalimnion (Low)-BOD Metalimnion (Low)-Light intensity (Medium)-DO epilimnion (Medium): High :5, Very Low :5, Medium :40, Low :50 |  |
|  |  |  |  |  | Chlorophyll-a metalimnion (Low)-BOD Metalimnion (Low)-Light intensity (Medium)-DO epilimnion (Low): High :5, Very Low :5, Medium :40, Low :50 |  |
|  |  |  |  |  | Chlorophyll-a metalimnion (Low)-BOD Metalimnion (Low)-Light intensity (Low)-DO epilimnion (High): High :5, Very Low :5, Medium :40, Low :50 |  |
|  |  |  |  |  | Chlorophyll-a metalimnion (Low)-BOD Metalimnion (Low)-Light intensity (Low)-DO epilimnion (Medium): High :5, Very Low :5, Medium :40, Low :50 |  |
|  |  |  |  |  | Chlorophyll-a metalimnion (Low)-BOD Metalimnion (Low)-Light intensity (Low)-DO epilimnion (Low): High :5, Very Low :5, Medium :40, Low :50 |  |
| Stocking density | Child | Expert knowledge | Interview with IWCC farmers and staffs of Fisheries Agency | Chance | Dry season:  High:20  Medium:70  Low:10 | High:11.7  Medium:45.0  Low:43.3 |
|  |  |  |  |  | Wet season:  High:10  Medium:40  Low:50 |  |
| Number of active cages | Child | Expert knowledge | Interview with staffs of Fisheries Agency and data from Fisheries Agency | Chance | Dry season:  Zero: 0, Low: 5  Medium: 10, High: 85 | Zero:4.16  Low:17.5  Medium:22.5  High: 55.8 |
|  |  |  |  |  | Wet season  Zero: 5, Low: 20  Medium: 25, High:50 |  |
| Feeding management | Parent | Data | Interview with IWCC farmers and Erlania *et al*. (2010) | Chance | Twice Floating:40  None:5  Twice emerged:10  One Floating:35  One emerged:10 | Twice Floating:40  None:5  Twice emerged:10  One Floating:35  One emerged:10 |
| Organic sediment run off | Child | Expert knowledge | Validated with Marganof (2007) and Ministry of Public Work report (2013) | Chance | Rainfall intensity (No rain):  High: 25, Medium: 25, Low: 50 | High: 33.8  Medium: 30.1  Low: 36.1 |
|  |  |  |  |  | Rainfall intensity (Light rain):  High: 30, Medium: 30, Low: 40 |  |
|  |  |  |  |  | Rainfall intensity (Moderate rain):  High: 40, Medium: 30, Low: 30 |  |
|  |  |  |  |  | Rainfall intensity (Heavy rain):  High: 50, Medium: 40, Low: 10 |  |
| Feed | Child | Expert knowledge | Based on the paper by Erlania,Prasetio, & Joni (2010) evaluated with interview results with IWCC farmers | Chance | Number of active cages (Zero)-Stocking density (Low-High)-Feeding management (None-Twice floating): High:0, Medium:0, Low:100 | High: 63.3  Medium: 12.9  Low: 23.8 |
|  |  |  |  |  | Number of active cages (Low)-Stocking density (High)-Feeding management (Twice floating): High:60, Medium:20, Low:20 |  |
|  |  |  |  |  | Number of active cages (Low)-Stocking density (High)-Feeding management (None): High:0, Medium:0, Low:100 |  |
|  |  |  |  |  | Number of active cages (Low)-Stocking density (High)-Feeding management (Twice emerge): High:60, Medium:20, Low:20 |  |
|  |  |  |  |  | Number of active cages (Low)-Stocking density (High)-Feeding management (One floating): High:10, Medium:10, Low:80 |  |
|  |  |  |  |  | Number of active cages (Low)-Stocking density (Medium)-Feeding management (Twice floating): High:25, Medium:15, Low:60 |  |
|  |  |  |  |  | Number of active cages (Low)-Stocking density (Medium)-Feeding management (One floating): High:10, Medium:10, Low:80 |  |
|  |  |  |  |  | Number of active cages (Low)-Stocking density (Medium)-Feeding management (One emerge): High:10, Medium:10, Low:80 |  |
|  |  |  |  |  | Number of active cages (Low)-Stocking density (Low)-Feeding management (Twice floating): High:10, Medium:25, Low:65 |  |
|  |  |  |  |  | Number of active cages (Low)-Stocking density (Low)-Feeding management (None): High:0, Medium:0, Low:100 |  |
|  |  |  |  |  | Number of active cages (Low)-Stocking density (Low)-Feeding management (Twice emerge): High:25, Medium:15, Low:60 |  |
|  |  |  |  |  | Number of active cages (Low)-Stocking density (Low)-Feeding management (One floating): High:10, Medium:20, Low:70 |  |
|  |  |  |  |  | Number of active cages (Medium)-Stocking density (Low-High)-Feeding management (None-Twice emerge): High:0-80, Medium:0-20, Low:0-100 |  |
|  |  |  |  |  | Number of active cages (High)-Stocking density (Low-High)-Feeding management (None-Twice emerge): High:0-100, Medium:0-20, Low:0-100 |  |
| Accumulated fish feed | Child | Expert knowledge | Based on the paper of Junaidi, Syandri, and Azrita (2014) |  | Feed (High)-Water current velocity (Fast)  High: 60, Low:40 | High: 57.8  Low: 42.2 |
|  |  |  |  |  | Feed (High)-Water current velocity (Medium)  High: 50, Low:50 |  |
|  |  |  |  |  | Feed (High)-Water current velocity (Slow)  High: 40, Low:60 |  |
|  |  |  |  |  | Feed (Medium)-Water current velocity (Fast)  High: 75, Low:25 |  |
|  |  |  |  |  | Feed (Medium)-Water current velocity (Medium)  High: 65, Low:35 |  |
|  |  |  |  |  | Feed (Medium)-Water current velocity (Slow)  High: 55, Low:45 |  |
|  |  |  |  |  | Feed (Low)-Water current velocity (Fast)  High: 95, Low:5 |  |
|  |  |  |  |  | Feed (Low)-Water current velocity (Medium)  High: 85, Low:15 |  |
|  |  |  |  |  | Feed (Low)-Water current velocity (Slow)  High: 75, Low:25 |  |
| *Gobiopterus* disappearance | Child | - |  |  |  |  |
| Mass fish kills | Child | - |  |  |  |  |

# S4 Table. Outcomes of the model

| Scenario | | Season | State | MFK | G.disappearance |
| --- | --- | --- | --- | --- | --- |
| 1a | Business as usual | Dry | Calm wind-No Rain-Wind Dir (N, E, NE) | 1.77 | 10.9 |
| 1a | Business as usual | Dry | Calm wind-No Rain-Wind Dir (others) | 5.21 | 10.9 |
| 1a | Business as usual | Dry | Calm wind-Light Rain-Wind Dir (N, E, NE) | 2.43 | 11.46 |
| 1a | Business as usual | Dry | Calm wind-Light Rain-Wind Dir others) | 5.43 | 11.46 |
| 1a | Business as usual | Dry | Calm wind-Moderate Rain-Wind Dir (N, E, NE) | 17 | 25.2 |
| 1a | Business as usual | Dry | Calm wind-Moderate Rain-Wind Dir (others) | 10.1 | 25.2 |
| 1a | Business as usual | Rainy | Strong wind-Moderate Rain-Wind Dir (N, E, NE) | 32 | 89 |
| 1a | Business as usual | Rainy | Strong wind-Moderate Rain-Wind Dir (others) | 85.5 | 89 |
| 1a | Business as usual | Rainy | Strong wind-Heavy Rain-Wind Dir (N, E, NE) | 55 | 60.6 |
| 1a | Business as usual | Rainy | Strong wind-Heavy Rain-Wind Dir (others) | 22.2 | 60.6 |
| 1a | Business as usual | Rainy | Gale-storm wind-Moderate Rain-Wind Dir (N, E, NE) | 47.9 | 54 |
| 1a | Business as usual | Rainy | Gale-storm wind-Moderate Rain-Wind Dir (others) | 20 | 54 |
| 1a | Business as usual | Rainy | Gale-storm wind-Heavy Rain-Wind Dir (N, E, NE) | 75.3 | 79.5 |
| 1a | Business as usual | Rainy | Gale-storm wind-Heavy Rain-Wind Dir (others) | 28.7 | 79.5 |
| 1b | Without IWCCF | Dry | Calm wind-No Rain-Wind Dir (N, E, NE) | 0 | 10.9 |
| 1b | Without IWCCF | Dry | Calm wind-No Rain-Wind Dir (others) | 0 | 10.9 |
| 1b | Without IWCCF | Dry | Calm wind-Light Rain-Wind Dir (N, E, NE) | 0 | 11.46 |
| 1b | Without IWCCF | Dry | Calm wind-Light Rain-Wind Dir others) | 0 | 11.46 |
| 1b | Without IWCCF | Dry | Calm wind-Moderate Rain-Wind Dir (N, E, NE) | 0 | 25.2 |
| 1b | Without IWCCF | Dry | Calm wind-Moderate Rain-Wind Dir (Others) | 0 | 25.2 |
| 1b | Without IWCCF | Rainy | Strong wind-Moderate Rain-Wind Dir (N, E, NE) | 0 | 89 |
| 1b | Without IWCCF | Rainy | Strong wind-Moderate Rain-Wind Dir (others) | 0 | 89 |
| 1b | Without IWCCF | Rainy | Strong wind-Heavy Rain-Wind Dir (N, E, NE) | 0 | 60.6 |
| 1b | Without IWCCF | Rainy | Strong wind-Heavy Rain-Wind Dir (others) | 0 | 60.6 |
| 1b | Without IWCCF | Rainy | Gale-storm wind-Moderate Rain-Wind Dir (N, E, NE) | 0 | 54 |
| 1b | Without IWCCF | Rainy | Gale-storm wind-Moderate Rain-Wind Dir (others) | 0 | 54 |
| 1b | Without IWCCF | Rainy | Gale-storm wind-Heavy Rain-Wind Dir (N, E, NE) | 0 | 79.5 |
| 1b | Without IWCCF | Rainy | Gale-storm wind-Heavy Rain-Wind Dir (others) | 0 | 79.5 |
| 1c | With 6,000 IWCCF | Dry | Calm wind-No Rain-Wind Dir (N, E, NE) | 1.77 | 10.9 |
| 1c | With 6,000 IWCCF | Dry | Calm wind-No Rain-Wind Dir (others) | 5.21 | 10.9 |
| 1c | With 6,000 IWCCF | Dry | Calm wind-Light Rain-Wind Dir (N, E, NE) | 2.43 | 11.46 |
| 1c | With 6,000 IWCCF | Dry | Calm wind-Light Rain-Wind Dir others) | 5.43 | 11.46 |
| 1c | With 6,000 IWCCF | Dry | Calm wind-Moderate Rain-Wind Dir (N, E, NE) | 17 | 25.2 |
| 1c | With 6,000 IWCCF | Dry | Calm wind-Moderate Rain-Wind Dir (Others) | 10.1 | 25.2 |
| 1c | With 6,000 IWCCF | Rainy | Strong wind-Moderate Rain-Wind Dir (N, E, NE) | 32 | 89 |
| 1c | With 6,000 IWCCF | Rainy | Strong wind-Moderate Rain-Wind Dir (others) | 85.5 | 89 |
| 1c | With 6,000 IWCCF | Rainy | Strong wind-Heavy Rain-Wind Dir (N, E, NE) | 55 | 60.6 |
| 1c | With 6,000 IWCCF | Rainy | Strong wind-Heavy Rain-Wind Dir (others) | 22.2 | 60.6 |
| 1c | With 6,000 IWCCF | Rainy | Gale-storm wind-Moderate Rain-Wind Dir (N, E, NE) | 47.9 | 54 |
| 1c | With 6,000 IWCCF | Rainy | Gale-storm wind-Moderate Rain-Wind Dir (others) | 20 | 54 |
| 1c | With 6,000 IWCCF | Rainy | Gale-storm wind-Heavy Rain-Wind Dir (N, E, NE) | 75.3 | 79.5 |
| 1c | With 6,000 IWCCF | Rainy | Gale-storm wind-Heavy Rain-Wind Dir (others) | 28.7 | 79.5 |
| 1d | With IWCCF and reduction of internal P loading | Dry | Calm wind-No Rain-Wind Dir (N, E, NE) | 2.72 | 4.56 |
| 1d | With IWCCF and reduction of internal P loading | Dry | Calm wind-No Rain-Wind Dir (others) | 1.6 | 4.56 |
| 1d | With IWCCF and reduction of internal P loading | Dry | Calm wind-Light Rain-Wind Dir (N, E, NE) | 2.27 | 5.21 |
| 1d | With IWCCF and reduction of internal P loading | Dry | Calm wind-Light Rain-Wind Dir others) | 3.23 | 5.21 |
| 1d | With IWCCF and reduction of internal P loading | Dry | Calm wind-Moderate Rain-Wind Dir (N, E, NE) | 16.2 | 18.8 |
| 1d | With IWCCF and reduction of internal P loading | Dry | Calm wind-Moderate Rain-Wind Dir (Others) | 13.9 | 18.8 |
| 1d | With IWCCF and reduction of internal P loading | Rainy | Strong wind-Moderate Rain-Wind Dir (N, E, NE) | 30.8 | 33.8 |
| 1d | With IWCCF and reduction of internal P loading | Rainy | Strong wind-Moderate Rain-Wind Dir (others) | 23.3 | 33.8 |
| 1d | With IWCCF and reduction of internal P loading | Rainy | Strong wind-Heavy Rain-Wind Dir (N, E, NE) | 71.9 | 72.9 |
| 1d | With IWCCF and reduction of internal P loading | Rainy | Strong wind-Heavy Rain-Wind Dir (others) | 35.8 | 51.4 |
| 1d | With IWCCF and reduction of internal P loading | Rainy | Gale-storm wind-Moderate Rain-Wind Dir (N, E, NE) | 35.3 | 38.3 |
| 1d | With IWCCF and reduction of internal P loading | Rainy | Gale-storm wind-Moderate Rain-Wind Dir (others) | 26.6 | 38.3 |
| 1d | With IWCCF and reduction of internal P loading | Rainy | Gale-storm wind-Heavy Rain-Wind Dir (N, E, NE) | 54.9 | 57.9 |
| 1d | With IWCCF and reduction of internal P loading | Rainy | Gale-storm wind-Heavy Rain-Wind Dir (others) | 40.5 | 57.9 |
| 1e | With IWCCF and aeration | Dry | Calm wind-No Rain-Wind Dir (N, E, NE) | 0.21 | 9.53 |
| 1e | With IWCCF and aeration | Dry | Calm wind-No Rain-Wind Dir (others) | 4.74 | 9.53 |
| 1e | With IWCCF and aeration | Dry | Calm wind-Light Rain-Wind Dir (N, E, NE) | 0.29 | 9.62 |
| 1e | With IWCCF and aeration | Dry | Calm wind-Light Rain-Wind Dir others) | 4.78 | 9.62 |
| 1e | With IWCCF and aeration | Dry | Calm wind-Moderate Rain-Wind Dir (N, E, NE) | 2.34 | 11.9 |
| 1e | With IWCCF and aeration | Dry | Calm wind-Moderate Rain-Wind Dir (Others) | 5.67 | 11.9 |
| 1e | With IWCCF and aeration | Rainy | Strong wind-Moderate Rain-Wind Dir (N, E, NE) | 9.6 | 20.1 |
| 1e | With IWCCF and aeration | Rainy | Strong wind-Moderate Rain-Wind Dir (others) | 8.86 | 20.1 |
| 1e | With IWCCF and aeration | Rainy | Strong wind-Heavy Rain-Wind Dir (N, E, NE) | 26 | 38.6 |
| 1e | With IWCCF and aeration | Rainy | Strong wind-Heavy Rain-Wind Dir (others) | 16.1 | 38.6 |
| 1e | With IWCCF and aeration | Rainy | Gale-storm wind-Moderate Rain-Wind Dir (N, E, NE) | 12.2 | 23.1 |
| 1e | With IWCCF and aeration | Rainy | Gale-storm wind-Moderate Rain-Wind Dir (others) | 10 | 23.1 |
| 1e | With IWCCF and aeration | Rainy | Gale-storm wind-Heavy Rain-Wind Dir (N, E, NE) | 40.1 | 54.5 |
| 1e | With IWCCF and aeration | Rainy | Gale-storm wind-Heavy Rain-Wind Dir (others) | 22.2 | 54.5 |
| 1f | With IWCCF and reduction of internal P loading and aeration | Dry | Calm wind-No Rain-Wind Dir (N, E, NE) | 0.2 | 3.23 |
| 1f | With IWCCF and reduction of internal P loading and aeration | Dry | Calm wind-No Rain-Wind Dir (others) | 1.59 | 3.23 |
| 1f | With IWCCF and reduction of internal P loading and aeration | Dry | Calm wind-Light Rain-Wind Dir (N, E, NE) | 0.3 | 3.33 |
| 1f | With IWCCF and reduction of internal P loading and aeration | Dry | Calm wind-Light Rain-Wind Dir others) | 1.63 | 3.33 |
| 1f | With IWCCF and reduction of internal P loading and aeration | Dry | Calm wind-Moderate Rain-Wind Dir (N, E, NE) | 2.32 | 5.61 |
| 1f | With IWCCF and reduction of internal P loading and aeration | Dry | Calm wind-Moderate Rain-Wind Dir (Others) | 2.52 | 5.61 |
| 1f | With IWCCF and reduction of internal P loading and aeration | Rainy | Strong wind-Moderate Rain-Wind Dir (N, E, NE) | 4.96 | 11.9 |
| 1f | With IWCCF and reduction of internal P loading and aeration | Rainy | Strong wind-Moderate Rain-Wind Dir (others) | 7.92 | 11.9 |
| 1f | With IWCCF and reduction of internal P loading and aeration | Rainy | Strong wind-Heavy Rain-Wind Dir (N, E, NE) | 15.3 | 20.3 |
| 1f | With IWCCF and reduction of internal P loading and aeration | Rainy | Strong wind-Heavy Rain-Wind Dir (others) | 8.21 | 20.3 |
| 1f | With IWCCF and reduction of internal P loading and aeration | Rainy | Gale-storm wind-Moderate Rain-Wind Dir (N, E, NE) | 9.56 | 13.8 |
| 1f | With IWCCF and reduction of internal P loading and aeration | Rainy | Gale-storm wind-Moderate Rain-Wind Dir (others) | 5.68 | 13.8 |
| 1f | With IWCCF and reduction of internal P loading and aeration | Rainy | Gale-storm wind-Heavy Rain-Wind Dir (N, E, NE) | 19.1 | 24.4 |
| 1f | With IWCCF and reduction of internal P loading and aeration | Rainy | Gale-storm wind-Heavy Rain-Wind Dir (others) | 9.84 | 24.4 |
| 2a | Business as usual | Dry | Calm wind-No Rain-Wind Dir (N, E, NE) | 2.02 | 12.2 |
| 2a | Business as usual | Dry | Calm wind-No Rain-Wind Dir (others) | 2.73 | 12.2 |
| 2a | Business as usual | Dry | Calm wind-Light Rain-Wind Dir (N, E, NE) | 2.27 | 12.3 |
| 2a | Business as usual | Dry | Calm wind-Light Rain-Wind Dir others) | 3.24 | 12.3 |
| 2a | Business as usual | Dry | Calm wind-Moderate Rain-Wind Dir (N, E, NE) | 16.3 | 25.3 |
| 2a | Business as usual | Dry | Calm wind-Moderate Rain-Wind Dir (Others) | 14 | 25.3 |
| 2a | Business as usual | Rainy | Strong wind-Moderate Rain-Wind Dir (N, E, NE) | 45.7 | 54 |
| 2a | Business as usual | Rainy | Strong wind-Moderate Rain-Wind Dir (others) | 36.5 | 54 |
| 2a | Business as usual | Rainy | Strong wind-Heavy Rain-Wind Dir (N, E, NE) | 56.6 | 79.6 |
| 2a | Business as usual | Rainy | Strong wind-Heavy Rain-Wind Dir (others) | 71.9 | 79.6 |
| 2a | Business as usual | Rainy | Gale-storm wind-Moderate Rain-Wind Dir (N, E, NE) | 52.5 | 89 |
| 2a | Business as usual | Rainy | Gale-storm wind-Moderate Rain-Wind Dir (others) | 41.7 | 89 |
| 2a | Business as usual | Rainy | Gale-storm wind-Heavy Rain-Wind Dir (N, E, NE) | 81.6 | 60.6 |
| 2a | Business as usual | Rainy | Gale-storm wind-Heavy Rain-Wind Dir (others) | 64 | 60.6 |
| 2b | Without IWCCF | Dry | Calm wind-No Rain-Wind Dir (N, E, NE) | 0 | 4.56 |
| 2b | Without IWCCF | Dry | Calm wind-No Rain-Wind Dir (others) | 0 | 4.56 |
| 2b | Without IWCCF | Dry | Calm wind-Light Rain-Wind Dir (N, E, NE) | 0 | 5.21 |
| 2b | Without IWCCF | Dry | Calm wind-Light Rain-Wind Dir others) | 0 | 5.21 |
| 2b | Without IWCCF | Dry | Calm wind-Moderate Rain-Wind Dir (N, E, NE) | 0 | 18.9 |
| 2b | Without IWCCF | Dry | Calm wind-Moderate Rain-Wind Dir (Others) | 0 | 18.9 |
| 2b | Without IWCCF | Rainy | Strong wind-Moderate Rain-Wind Dir (N, E, NE) | 0 | 47.5 |
| 2b | Without IWCCF | Rainy | Strong wind-Moderate Rain-Wind Dir (others) | 0 | 47.5 |
| 2b | Without IWCCF | Rainy | Strong wind-Heavy Rain-Wind Dir (N, E, NE) | 0 | 73 |
| 2b | Without IWCCF | Rainy | Strong wind-Heavy Rain-Wind Dir (others) | 0 | 73 |
| 2b | Without IWCCF | Rainy | Gale-storm wind-Moderate Rain-Wind Dir (N, E, NE) | 0 | 54.1 |
| 2b | Without IWCCF | Rainy | Gale-storm wind-Moderate Rain-Wind Dir (others) | 0 | 54.1 |
| 2b | Without IWCCF | Rainy | Gale-storm wind-Heavy Rain-Wind Dir (N, E, NE) | 0 | 82.5 |
| 2b | Without IWCCF | Rainy | Gale-storm wind-Heavy Rain-Wind Dir (others) | 0 | 82.5 |
| 2c | 6,000 IWCCF (no reduction of internal P action, and current feeding management) | Dry | Calm wind-No Rain-Wind Dir (N, E, NE) | 1.77 | 10.9 |
| 2c | 6,000 IWCCF (no reduction of internal P action, and current feeding management) | Dry | Calm wind-No Rain-Wind Dir (others) | 5.21 | 10.9 |
| 2c | 6,000 IWCCF (no reduction of internal P action, and current feeding management) | Dry | Calm wind-Light Rain-Wind Dir (N, E, NE) | 2.43 | 11.46 |
| 2c | 6,000 IWCCF (no reduction of internal P action, and current feeding management) | Dry | Calm wind-Light Rain-Wind Dir others) | 5.43 | 11.46 |
| 2c | 6,000 IWCCF (no reduction of internal P action, and current feeding management) | Dry | Calm wind-Moderate Rain-Wind Dir (N, E, NE) | 17 | 25.2 |
| 2c | 6,000 IWCCF (no reduction of internal P action, and current feeding management) | Dry | Calm wind-Moderate Rain-Wind Dir (Others) | 10.1 | 25.2 |
| 2c | 6,000 IWCCF (no reduction of internal P action, and current feeding management) | Rainy | Strong wind-Moderate Rain-Wind Dir (N, E, NE) | 47.9 | 47.4 |
| 2c | 6,000 IWCCF (no reduction of internal P action, and current feeding management) | Rainy | Strong wind-Moderate Rain-Wind Dir (others) | 36.4 | 47.4 |
| 2c | 6,000 IWCCF (no reduction of internal P action, and current feeding management) | Rainy | Strong wind-Heavy Rain-Wind Dir (N, E, NE) | 55 | 60.6 |
| 2c | 6,000 IWCCF (no reduction of internal P action, and current feeding management) | Rainy | Strong wind-Heavy Rain-Wind Dir (others) | 22.2 | 60.6 |
| 2c | 6,000 IWCCF (no reduction of internal P action, and current feeding management) | Rainy | Gale-storm wind-Moderate Rain-Wind Dir (N, E, NE) | 47.9 | 54 |
| 2c | 6,000 IWCCF (no reduction of internal P action, and current feeding management) | Rainy | Gale-storm wind-Moderate Rain-Wind Dir (others) | 20 | 54 |
| 2c | 6,000 IWCCF (no reduction of internal P action, and current feeding management) | Rainy | Gale-storm wind-Heavy Rain-Wind Dir (N, E, NE) | 75.3 | 79.5 |
| 2c | 6,000 IWCCF (no reduction of internal P action, and current feeding management) | Rainy | Gale-storm wind-Heavy Rain-Wind Dir (others) | 28.7 | 79.5 |
| 2d | 6,000 IWCCF (better feeding management and internal P loading control) | Dry | Calm wind-No Rain-Wind Dir (N, E, NE) | 0.85 | 0.85 |
| 2d | 6,000 IWCCF (better feeding management and internal P loading control) | Dry | Calm wind-No Rain-Wind Dir (others) | 0.85 | 0.85 |
| 2d | 6,000 IWCCF (better feeding management and internal P loading control) | Dry | Calm wind-Light Rain-Wind Dir (N, E, NE) | 1.22 | 1.22 |
| 2d | 6,000 IWCCF (better feeding management and internal P loading control) | Dry | Calm wind-Light Rain-Wind Dir others) | 1.22 | 1.22 |
| 2d | 6,000 IWCCF (better feeding management and internal P loading control) | Dry | Calm wind-Moderate Rain-Wind Dir (N, E, NE) | 8.83 | 8.83 |
| 2d | 6,000 IWCCF (better feeding management and internal P loading control) | Dry | Calm wind-Moderate Rain-Wind Dir (Others) | 8.83 | 8.83 |
| 2d | 6,000 IWCCF (better feeding management and internal P loading control) | Rainy | Strong wind-Moderate Rain-Wind Dir (N, E, NE) | 24.8 | 24.8 |
| 2d | 6,000 IWCCF (better feeding management and internal P loading control) | Rainy | Strong wind-Moderate Rain-Wind Dir (others) | 24.8 | 24.8 |
| 2d | 6,000 IWCCF (better feeding management and internal P loading control) | Rainy | Strong wind-Heavy Rain-Wind Dir (N, E, NE) | 39 | 39 |
| 2d | 6,000 IWCCF (better feeding management and internal P loading control) | Rainy | Strong wind-Heavy Rain-Wind Dir (others) | 39 | 39 |
| 2d | 6,000 IWCCF (better feeding management and internal P loading control) | Rainy | Gale-storm wind-Moderate Rain-Wind Dir (N, E, NE) | 28.5 | 28.5 |
| 2d | 6,000 IWCCF (better feeding management and internal P loading control) | Rainy | Gale-storm wind-Moderate Rain-Wind Dir (others) | 28.5 | 28.5 |
| 2d | 6,000 IWCCF (better feeding management and internal P loading control) | Rainy | Gale-storm wind-Heavy Rain-Wind Dir (N, E, NE) | 44.2 | 44.2 |
| 2d | 6,000 IWCCF (better feeding management and internal P loading control) | Rainy | Gale-storm wind-Heavy Rain-Wind Dir (others) | 44.2 | 44.2 |
